# Supplementary material for: Use of GLP1 receptor agonists in early pregnancy and reproductive safety: a multicentre, observational, prospective cohort study based on the databases of six Teratology Information Services
Source: BMJ Open. 2024 Apr 24;14(4):e083550. doi: 10.1136/bmjopen-2023-083550 (PMC11043712; doi:10.1136/bmjopen-2023-083550)
Supplement: Supplementary data [file bmjopen-2023-083550supp001.pdf]

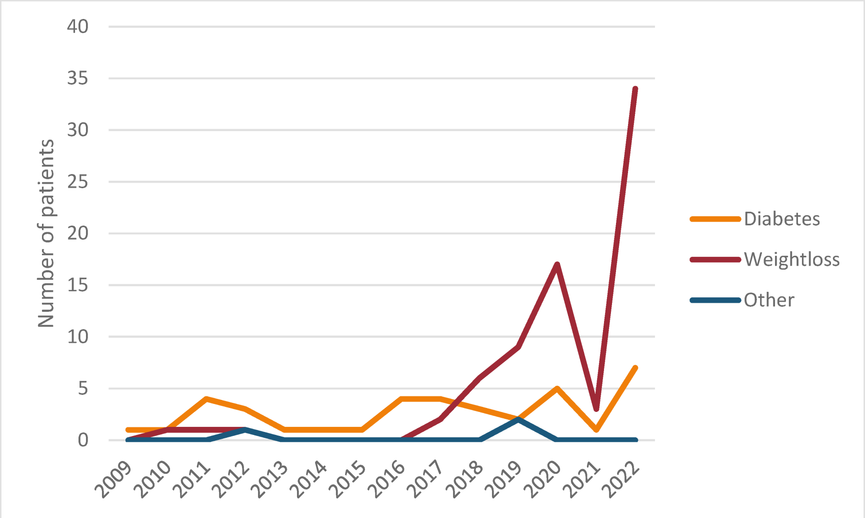

Supplemental Figure 1: Evolution of the clinical indications for glucagon-like peptide 1 receptor agonists
