# Supplementary material for: Use of GLP1 receptor agonists in early pregnancy and reproductive safety: a multicentre, observational, prospective cohort study based on the databases of six Teratology Information Services
Source: BMJ Open. 2024 Apr 24;14(4):e083550. doi: 10.1136/bmjopen-2023-083550 (PMC11043712; doi:10.1136/bmjopen-2023-083550)
Supplement: Supplementary data [file bmjopen-2023-083550supp002.pdf]

Supplemental Table 1 Major birth defects in GLP1-RA and the diabetic and overweight/obese reference groups, concomitant medications, and maternal conditions

Major birth defects in GLP1-RA exposed pregnancies	Outcome	GA at birth / pregnancy loss§	GLP1-RA (dose) / indication	Exposure§	Concomitant medications and pre-/pregnancy supplements (GA of exposure)	Maternal conditions
Ventricular septal defect	Live birth	38	Liraglutide (unknown) / Weight loss	0-6	folate (6-12)	Obesity (BMI 34 kg/m^2), gestational diabetes
Ectopic kidney	Live birth	38	Semaglutide (1 mg/wk) / Weight loss	0-4	methylphenidate (until week 6), folate (started before pregnancy)	Endometriosis, ADHD, alcohol (< 1 drink/day, timing unknown), BMI 25 kg/m^2
Multiple (brain, hands and heart) anomalies (nothing was found post mortem to suggest genetic or cytogenetic finding)	TOP	17	Liraglutide (unknown) / Weight loss	0-5	folate (started before pregnancy)	Overweight (BMI 27 kg/m^2)
**Major birth defects in diabetic patients pregnancies**	**Outcome**		**Antidiabetic drugs / indication**		**Concomitant medications and pre-/pregnancy supplements (GA of exposure)**	**Maternal conditions**
Club foot	Live birth	38	Metformin 1700 mg/d diabetes	0-7	folate (started before pregnancy), aluminium hydroxide*	Borderline personality, polycystic ovaries, obesity (BMI 40 kg/m^2)
Polymalformative syndrome (holoprosencephaly, rib deformity, corpus callosum agenesis, patent foramen ovale, hemivertebra, renal agenesis, spine malformation, ventricular septal defect, congenital disorder of glycosylation)	Live birth	40	Sitagliptin 100 mg/d, dapagliflozine, insulin diabetes	0-6<br>0-6<br>6-40	folate (started at 6 weeks), levothyroxine (0-40)	obesity (BMI 40 kg/m^2), hypothyroidism, tobacco (< 5 cig/day)
Spina bifida & other anomalies typical of diabetes	TOP	18	Metformin 2550 mg/d, glimepiride, insuline, diabetes	0-6<br>0-5<br>0-40	losartan (0-5), simvastatin (0-5)	Multiple sclerosis, arterial hypertensio

						n, obesity (BMI 34 kg/m^2)
**Major birth defects in overweight/obese patients pregnancies**	**Outcome**				**Concomitant medications**	**Maternal conditions**
Tetralogy of Fallot	Live birth	36			paracetamol (0-40), dipyrone*, passiflora valerian*, thiamine*, pyridoxine*, folate (started before pregnancy)	Overweight (BMI 28 kg/m^2)
Double outlet ventricle	TOP	17			escitalopram (0-17) acetylsalicylic acid 1000 mg*, folate (started before pregnancy), dental X-ray (6)	Obesity (BMI 30 kg/m^2), migraine
Acrania	TOP	15			sulpiride (0-15), acetazolamide oral (0-15), esomeprazole (0-15), folate (started before pregnancy)	Obesity (BMI 46 kg/m^2), schizophrenia
Biliary atresia	TOP	22			thyroxine (4-22), cefuroxime (7-8), influenza vaccination (14), folate (from beginning of pregnancy)	Obesity (BMI 36 kg/m^2), hypothyroidism
Oligohydramnios on early detailed U/S, urinary bladder distention/dilatation, compatible with posterior urethral valve	TOP	17			budesonide and formoterol (6-17), folate (started before pregnancy	Overweight (BMI 29 kg/m^2), asthma
* sometime in pregnancy, § weeks of gestation, GA : gestational age; TOP: termination of pregnancy						
