# Supplementary material for: Use of GLP1 receptor agonists in early pregnancy and reproductive safety: a multicentre, observational, prospective cohort study based on the databases of six Teratology Information Services
Source: BMJ Open. 2024 Apr 24;14(4):e083550. doi: 10.1136/bmjopen-2023-083550 (PMC11043712; doi:10.1136/bmjopen-2023-083550)
Supplement: Author's manuscript [file bmjopen-2023-083550.draft_revisions.pdf]

BMJ Open is committed to open peer review. As part of this commitment we make the peer review history of every article we publish publicly available.

When an article is published we post the peer reviewers' comments and the authors' responses online. We also post the versions of the paper that were used during peer review. These are the versions that the peer review comments apply to.

The versions of the paper that follow are the versions that were submitted during the peer review process. They are not the versions of record or the final published versions. They should not be cited or distributed as the published version of this manuscript.

BMJ Open is an open access journal and the full, final, typeset and author-corrected version of record of the manuscript is available on our site with no access controls, subscription charges or pay-per-view fees (http://bmjopen.bmj.com).

If you have any questions on BMJ Open's open peer review process please email info.bmjopen@bmj.com

BMJ Open

Reproductive safety of glucagon-like peptide 1 receptor agonists (GLP1-RA) after maternal exposure in early pregnancy: a comparative ENTIS cohort study

Journal:	*BMJ Open*
Manuscript ID	bmjopen-2023-083550
Article Type:	Original research
Date Submitted by the Author:	22-Dec-2023

Complete List of Authors:	Dao, Kim ; Centre Hospitalier Universitaire Vaudois (CHUV) and University of Lausanne, Swiss Teratogen Information Service and Clinical Pharmacology Service<br>Shechtman, Svetlana; Ministry of Health, The Israeli Teratology Information Service<br>Weber-Schoendorfer, Corinna; Charité Universitätsmedizin Berlin, , Pharmakovigilanzzentrum Embryonaltoxikologie, Institut für Klinische Pharmakologie und Toxikologie<br>Diav-Citrin, Orna ; Ministry of Health, The Israeli Teratology Information Service; Hebrew University of Jerusalem Hadassah Medical School<br>Murad, Reem; Ministry of Health, The Israeli Teratology Information Service<br>Berlin, Maya; Shamir Medical Center Assaf Harofeh, Clinical Pharmacology and Toxicology Unit, Drug Consultation Center, Zerifin TIS, affiliated with the Faculty of Medicine, Tel Aviv University<br>Hazan, Ariela; Shamir Medical Center Assaf Harofeh, Clinical Pharmacology and Toxicology Unit, Drug Consultation Center, Zerifin TIS, affiliated with the Faculty of Medicine, Tel Aviv University<br>Richardson, Jonathan L; Newcastle Upon Tyne Hospitals NHS Foundation Trust, The UK Teratology Information Service<br>Eleftheriou, Georgios; Hospital ASST Papa Giovanni XXIII, Poison Control Center<br>Rousson, Valentin; University of Lausanne, Center for Primary Care and Public Health<br>Diezi, Leonore; Centre Hospitalier Universitaire Vaudois (CHUV) and University of Lausanne, Swiss Teratogen Information Service and Clinical Pharmacology Service<br>Haefliger, David; Centre Hospitalier Universitaire Vaudois (CHUV) and University of Lausanne, Swiss Teratogen Information Service and Clinical Pharmacology Service<br>Simões-Wüst, Ana Paula ; University Hospital Zurich, University of Zurich, Department of Obstetrics<br>Addor, Marie-Claude; Centre Hospitalier Universitaire Vaudois (CHUV), Department of Woman-Mother-Child<br>Baud, David; Centre Hospitalier Universitaire Vaudois (CHUV) and University of Lausanne, Materno-Fetal and Obstetrics Research Unit, Department Woman-Mother-Child<br>Lamine, Faiza; Centre Hospitalier Universitaire Vaudois, Endocrinology, Diabetes and Metabolism Service; Hôpital Riviera-Chablais,

Endocrinology and Diabetes Unit. Internal Medicine Service.
Panchaud, Alice; University of Bern, Institute of Primary Health Care (BIHAM); Centre Hospitalier Universitaire Vaudois, Service of Pharmacy
Buclin, Thierry; Centre Hospitalier Universitaire Vaudois (CHUV) and University of Lausanne, Swiss Teratogen Information Service and Clinical Pharmacology Service
Girardin, François; Centre Hospitalier Universitaire Vaudois (CHUV) and University of Lausanne, Swiss Teratogen Information Service and Clinical Pharmacology Service
Winterfeld, Ursula; Centre Hospitalier Universitaire Vaudois (CHUV) and University of Lausanne, Swiss Teratogen Information Service and Clinical Pharmacology Service

Keywords: Pregnant Women, Safety, Diabetes in pregnancy < DIABETES & ENDOCRINOLOGY, Obesity

SCHOLARONE™
Manuscripts

I, the Submitting Author has the right to grant and does grant on behalf of all authors of the Work (as defined in the below author licence), an exclusive licence and/or a non-exclusive licence for contributions from authors who are: i) UK Crown employees; ii) where BMJ has agreed a CC-BY licence shall apply, and/or iii) in accordance with the terms applicable for US Federal Government officers or employees acting as part of their official duties; on a worldwide, perpetual, irrevocable, royalty-free basis to BMJ Publishing Group Ltd ("BMJ") its licensees and where the relevant Journal is co-owned by BMJ to the co-owners of the Journal, to publish the Work in this journal and any other BMJ products and to exploit all rights, as set out in our licence.

The Submitting Author accepts and understands that any supply made under these terms is made by BMJ to the Submitting Author unless you are acting as an employee on behalf of your employer or a postgraduate student of an affiliated institution which is paying any applicable article publishing charge ("APC") for Open Access articles. Where the Submitting Author wishes to make the Work available on an Open Access basis (and intends to pay the relevant APC), the terms of reuse of such Open Access shall be governed by a Creative Commons licence – details of these licences and which Creative Commons licence will apply to this Work are set out in our licence referred to above.

Other than as permitted in any relevant BMJ Author's Self Archiving Policies, I confirm this Work has not been accepted for publication elsewhere, is not being considered for publication elsewhere and does not duplicate material already published. I confirm all authors consent to publication of this Work and authorise the granting of this licence.

For peer review only

Reproductive safety of glucagon-like peptide 1 receptor agonists (GLP1-RA) after maternal exposure in early pregnancy: a comparative ENTIS cohort study

Kim Dao, MD[1], Svetlana Shechtman, PhD[2], Corinna Weber-Schoendorfer, MD-PhD[3], Orna Diav-Citrin, MD[2,4], Reem Hegla Murad[2], Maya Berlin, PhD, BSCPharm[5], Ariela Hazan Bsc[5], Jonathan Luke Richardson, PhD[6], Georgios Eleftheriou, MD-PhD[7], Valentin Rousson, PhD[8], Leonore Diezi[1], MD, David Haefliger[1], MD, Ana Paula Simoes-Wüst[9], PhD, Marie-Claude Addor, MD[10], David Baud, MD-PhD[11], Faiza Lamine[12] MD, Alice Panchaud[13,14], PhD, Thierry Buclin, MD-PhD[1], François Girardin, MD[1*], Ursula Winterfeld, PhD[1*]

[1] Swiss Teratogen Information Service and Clinical Pharmacology Service, Centre Hospitalier Universitaire Vaudois (CHUV) and University of Lausanne, Lausanne, Switzerland

[2] The Israeli Teratology Information Service, Ministry of Health, Jerusalem, Israel

[3] Charité - Universitätsmedizin Berlin, Pharmakovigilanzzentrum Embryonaltoxikologie, Institut für Klinische Pharmakologie und Toxikologie, Berlin, Germany

[4] The Hebrew University and Hadassah Medical School, Jerusalem, Israel

[5] Clinical Pharmacology and Toxicology Unit, Drug Consultation Center, Shamir Medical Center (Assaf Harofeh), Zerifin TIS, affiliated with the Faculty of Medicine, Tel Aviv University, Tel Aviv, Israel

[6] The UK Teratology Information Service, Newcastle upon Tyne Hospitals NHS Foundation Trust, Newcastle Upon Tyne, UK

[7] Poison Control Center, Hospital ASST Papa Giovanni XXIII, Bergamo, Italy

[8] Center for Primary Care and Public Health, University of Lausanne, Switzerland

[9] Department of Obstetrics, University Hospital Zurich, University of Zurich, Switzerland

[10] Department of Woman-Mother-Child, Centre Hospitalier Universitaire Vaudois (CHUV), Lausanne, Switzerland

[11] Materno-Fetal and Obstetrics Research Unit, Department Woman-Mother-Child, Centre Hospitalier Universitaire Vaudois (CHUV) and University of Lausanne, Lausanne, Switzerland

[12] Endocrinology, Diabetes and Metabolism Service. Centre Hospitalier Universitaire Vaudois (CHUV), Lausanne, Switzerland. Endocrinology and Diabetes Unit. Internal Medicine Service. Hôpital Riviera Chablais, Rennaz, Switzerland.

[13] Institute of Primary Health Care (BIHAM), University of Bern, Bern, Switzerland

[14] Service of Pharmacy, Centre Hospitalier Universitaire Vaudois (CHUV), Lausanne, Switzerland

* contributed equally

Journal: **BMJ Open** as an **Original Contribution**

Word count: 3581

For peer review only

Corresponding author

Dr. Ursula Winterfeld

Swiss Teratology Information Service (STIS) and Clinical Pharmacology Service, Lausanne University Hospital and University of Lausanne, Lausanne, Switzerland

e-mail: ursula.winterfeld@chuv.ch

Tel: +41-21-3144267

Competing interests

None declared

Funding

This work was supported by Swiss National Science Foundation grant number SNSF 320030_214844

Acknowledgement: The authors would like to thank pharmacists Sarit Carmi and Shira Shalit, from the Division of Clinical Pharmacy, Institute for Drug Research, School of Pharmacy, Hebrew University and Hadassah Medical school, Jerusalem, Israel, for their valuable contribution to the data collection, done while acquiring their PharmD degree. We thank the whole team at STIS for their support, in particular the computer scientist Mr. F. Veuve.

Data Availability Statement

Source data related to the cohort study are available on https://zenodo.org

For peer review only

Abstract

Objectives: Glucagon-like peptide 1 receptor agonists (GLP1-RA) are indicated for the treatment of type 2 diabetes and more recently for weight loss. The aim of this study was to assess the risks associated with GLP1-RA exposure during early pregnancy.

Design: This multicentre, observational prospective cohort study compared pregnancy outcomes in women exposed to a GLP1-RA in early pregnancy either for diabetes or obesity treatment with those in two reference groups: (i) of diabetic women exposed to at least one non-GLP1-RA antidiabetic drug during the first trimester and (ii) a reference group of overweight/obese women without diabetes, between 2009 and 2022.

Setting: Data were collected from the databases of six Teratology Information Services.

Participants: This study included 168 pregnancies of women exposed to a GLP1-RA during the first trimester, alongside a reference group of 156 pregnancies of women with diabetes and 163 pregnancies of overweight/obese women.

Results: Exposure to a GLP1-RA in the first trimester was not associated with a risk of major birth defects when compared to diabetes (2.6% versus 2.3%; adjusted odds ratio (OR) [95% confidence interval], 0.78 [0.13–4.83]) or to overweight/obese (2.6% versus 3.9%; adjusted OR 0.63 [0.13–3.07]). For the GLP1-RA group, cumulative incidence for live births, pregnancy losses, and pregnancy terminations was 58%, 22%, and 18%, respectively. In the diabetes reference group, corresponding estimates were 64%, 26%, and 6%, while in the overweight/obese reference group, they were 63%, 28%, and 8%. Cox proportional cause-specific hazards models indicated no increased risk of pregnancy losses in the GLP1-RA versus the diabetes and the overweight/obese reference groups, in both crude and adjusted analyses.

Conclusions: This study offers reassurance in cases of inadvertent exposure to GLP1-RA during the first trimester of pregnancy. Due to the limited sample size, larger studies are required to validate these findings.

For peer review only

Keywords: Glucagon-like peptide 1 receptor agonists, liraglutide, semaglutide, dulaglutide, exenatide pregnancy, reproductive safety.

Strengths and limitations of this study

- This observational prospective multicentre study aimed to assess the reproductive safety of early pregnancy exposure to GLP1-RA, providing valuable insights into a previously underexplored area.
- The study design incorporated two reference groups (diabetes and overweight/obese), to reduce the influence of potential confounding variables.
- GLP1-RA were analysed as a single homogeneous group, only allowing for the exploration of potential class effects as specific GLP1-RA had limited individual exposure.
- The availability of additional data, such as glycated haemoglobin or fasting glucose levels, varied within the study sample, which affected our ability to precisely describe disease severity among patients with diabetes.

Introduction

Glucagon-like peptide 1 receptor agonists (GLP1-RA) have been widely used as therapeutic agents for the management of type 2 diabetes. More recently, certain GLP1-RA (liraglutide, semaglutide) have also gained approval in some countries for the treatment of obesity. The demand for GLP1-RA approved for obesity is high, so that there have even been supply shortages in Europe [1]. Since the prevalence of both overweight and obesity among women of childbearing age is increasing substantially in most countries (between 30% in some European countries and nearly 50% in the USA), it is to be expected that a further growing number of women of reproductive age will be treated with a GLP1-RA [2-4]. Approximately 50% of pregnancies worldwide are unplanned [5], therefore the question of the safety of GLP1-RA is particularly relevant.

This drug class includes short acting GLP1-RA exenatide, lixisenatide and beinaglutide, as well as long-acting GLP1 RA dulaglutide, semaglutide, liraglutide and albiglutide (discontinued in 2017 at the request of the marketing-authorisation holder due to steady decline in sales). By mimicking the actions

For peer review only

of the hormone glucagon-like peptide 1, these agents improve blood glycaemic control by increasing glucose-dependent insulin secretion, decreasing inappropriate glucagon secretion, and regulate appetite (by increasing satiety and delaying gastric emptying) to promote weight loss. The safety profile of GLP1-RA use during the first trimester of pregnancy has been investigated in previous studies, which did not reveal an elevated risk of major birth defects. One single case of exposure to liraglutide in the first trimester has been reported, describing a favourable outcome for the newborn [6]. A registry for exenatide documented seven cases of exposure during pregnancy, but follow-up information is lacking [7]. A case report presents the outcomes of two pregnancies in a 40-year-old woman with diabetes and obesity undergoing exenatide treatment, describing the birth of one healthy child and another with an atrial septal defect that spontaneously resolved by the age of three [8]. One single case of exposure to semaglutide in early pregnancy due to off-label treatment for polycystic ovary syndrome was reported with no birth defect [9]. Another case of exposure to dulaglutide during first trimester of pregnancy for the treatment of type 2 diabetes did not result in any reported birth defects [10]. Due to paucity of data concerning semaglutide and human pregnancy outcomes, the manufacturer advises stopping this treatment two months before conception [11]. In a multinational population-based cohort study involving 51'826 pregnant women diagnosed with type 2 diabetes and their infants, the standardized prevalence of major congenital malformations was 8.3% among infants with periconceptional exposure to GLP1-RA (A10BJ) (n = 938) [12]. Compared with insulin, there was no increased risk of major congenital malformations (adjusted RR 0.95 95% CI, 0.72-1.26) for infants exposed to GLP1-RA.

According to product labelling, animal studies have indicated a potential for reproductive toxicity at maternally toxic doses for semaglutide, dulaglutide, exenatide and liraglutide. An increased risk of malformations was observed for liraglutide and semagutide at doses comparable to those administered in humans [13-17]. GLP1-RA are characterised by their large molar mass, ranging from 3700 g/mol (liraglutide) to 4100 g/mol (semaglutide and exenatide) and even up to 63,000 g/mol

For peer review only

(dulaglutide). Even though additional physico-chemical properties play important roles, placental transfer of drugs with such high molecular size is generally not anticipated in the first trimester of pregnancy, unless a specific mechanism for transfer exists [18].

While previous studies did not detect an increased risk of major birth defects following the use of GLP1-RA during the first trimester of pregnancy, further confirmation from additional studies is warranted. The primary objective of this study was to prospectively evaluate the risk of major birth defects, spontaneous pregnancy losses (including abortions and stillbirths) and pregnancy terminations following first-trimester exposure to a GLP1-RA. Secondary objectives included describing additional pregnancy outcomes, such as gestational weeks at birth, birth weight, and categorical neonatal outcomes like preterm birth and small or large for gestational age.

Material & Methods

Study design and dataset description

This multicentre, prospective, observational cohort study was conducted involving six participating centres that are members of the European Network of Teratology Information Services (ENTIS) in five countries: Germany, Israel, Italy, Switzerland, and the United Kingdom. ENTIS is an organisation of specialised services providing expertise on potential risks associated with medication exposure during pregnancy and breastfeeding at an individual level [19]. Standardised protocols are followed for data collection at each participating centre [20]. Methodological aspects pertaining to collaborative studies of a similar nature have been extensively documented in the existing literature, thus serving as a reference for performing the present study [21].

Health professionals and pregnant women spontaneously contact a Teratology Information Service (TIS) for a risk assessment during pregnancy. Data collection is performed at this first contact with the TIS and following the anticipated date of delivery. Standardised questionnaires are used, administered to the patient and/or their health care provider. Maternal characteristics, such as age, tobacco use and

For peer review only

alcohol consumption, as well as medical and obstetric history, are recorded. Detailed information regarding drug exposure and drug treatment indication, including dose, timing of therapy initiation, duration and concurrent medications, is also documented during the initial contact with the TIS. After the expected date of delivery, follow-up is conducted through structured mailed questionnaires and/or a structured telephone interview. The follow-up process seeks information on various aspects, including further medication use, pregnancy outcomes, gestational age at delivery, birth weight, presence of birth defects, and neonatal complications. For this study, additional data pertaining to diabetes severity and obesity were also collected, where available. Socioeconomic and educational data were mostly not recorded [22].

Exposed and reference groups

The study sample was comprised of women exposed to any GLP1-RA, and two reference groups: one of diabetic women, the other of overweight/obese women without diabetes. The patients, or their healthcare providers, contacted one of the six participating TIS between 2009 and 2022. Only patients with prospectively ascertained pregnancy outcomes, meaning those with unknown pregnancy outcomes or prenatal pathologies at the time of study enrolment, were included. The GLP1-RA exposed group consisted of pregnant women who used at least one GLP1-RA (identified by ATC codes A10BJ, A10AE54 or A10AE56) either as monotherapy or in combination with other medications during the first trimester of pregnancy.

The first reference group included pregnancies of patients diagnosed with pre-existing diabetes mellitus (ICD-10 codes E10-E13), who were exposed to non-GLP1-RA antidiabetic drugs during the first trimester of pregnancy (in most cases metformin). Patient selection for this reference group was done through a randomised process, choosing from the same prospective cohort within the corresponding TIS and within the same time frame of contact (±3 year). The second reference group primarily included pregnancies of patients classified as overweight (BMI ≥ 25 kg/m2) or obese (BMI ≥ 30 kg/m2) (ICD-10 code E66). They were aimed to be matched for BMI with the exposed group for a BMI of ± 2 kg/m^2.

For peer review only

Similarly, patient selection within this reference group was randomised from the same prospective cohort within the corresponding TIS and within the same time frame of contact (±3 year).

Exclusion criteria for all three groups included exposure to any teratogenic drugs such as systemic retinoids (including acitretin, alitretinoin, bexarotene, isotretinoin, tretinoin), cytotoxic agents, selected antiepileptic drugs (including valproate, carbamazepine, phenytoin, fosphenytoin, primidone, topiramate, phenobarbital), thalidomide, leflunomide, lenalidomide, coumarin derivatives (including dicoumarol, phenindione, warfarin, phenprocoumon, acenocoumarol, fluindione), lithium, misoprostol, carbimazole, and methimazole/thiamazole at any time during pregnancy. Additionally, exclusion criteria encompassed exposure to angiotensin-converting enzyme inhibitors, angiotensin II receptor blockers, or tetracyclines during the second and third trimesters, presence of malignancies or malignancy-related conditions, multiple pregnancies, and duplicate cases. Data sent from different TIS were analysed anonymously [22].

Outcomes

Birth defects were classified by two independent co-authors (MCA, DB) who were blinded to exposure data. The classification was performed using the European Network of Population-based Registries for the Epidemiological Surveillance of Congenital Anomalies (EUROCAT) ICD10-BPA system [23]. The assessment of major birth defects was restricted to live births and pregnancy losses with confirmed outcomes after appropriate medical examination, ensuring reliable knowledge on birth defect status. Birth defects with genetic or chromosomal anomalies and those occurring due to intrauterine infections were excluded. Due to significant likelihood of underreporting, minor birth defects were not included in the evaluation. Primary outcomes also included the risks of spontaneous pregnancy losses, which comprised both abortions and stillbirths as a combined outcome, categorised based on gestational age as either ≤22 weeks or >22 weeks, respectively. Additionally, the risks of pregnancy terminations were analysed. Secondary outcomes were preterm birth (<37 weeks of gestation after LMP), gestational age at birth and birth weight. Large (LGA) and small (SGA) for gestational age were

For peer review only

also compared between cohorts. LGA was defined as a birthweight exceeding the 90th percentile, and SGA as birthweight less than the 10th percentile based on WHO infant sex-specific growth charts [24].

Statistical Analysis

Crude risks of major birth defects were determined by dividing the total number of infants or fetuses with birth defects by the sum of all live-born infants, plus the number of cases with known birth defects in stillbirths and terminated pregnancies [19]. To assess the association between GLP1-RA exposure and the risk of major birth defects, multivariate logistic regression analysis was conducted, generating an odds ratio (OR) with a corresponding 95% confidence interval (CI) and adjusted for confounding factors, including maternal age, number of previous pregnancies and polymedication [19]. The number of previous pregnancies was categorised (0, 1, ≥2), and polymedication was defined as the use of more than one drug, including any other medication. An additional category for missing values was included to account for instances where data on any of the confounding factors, such as maternal age, history of previous pregnancies and polymedication, were not available [7].

Elective pregnancy terminations for personal reasons (ETOP) and elective terminations for medical reasons (MTOP), as well as spontaneous pregnancy losses (miscarriages and stillbirths), were treated as competing events in this study. The frequency of these outcomes was represented using cumulative incidence functions [25], while delayed entries were treated as described by Rousson et al. [26]. Women were thus considered to be at risk of experiencing one of these outcomes solely from their gestational age at the time of contact with the TIS. This allowed us to take into account the gestational age at the time of TIS contact, acknowledging that it was not independent of the outcome, given that certain pregnancy terminations may not be feasible at an advanced gestational age. Cases with missing information on gestational age at call or pregnancy outcome were excluded from the cumulative incidence analysis. To assess the association between exposure and the overall risk of pregnancy loss, cause-specific Cox proportional hazards models were used, considering any variation in gestational age at enrolment across the three groups. In the hazard models, adjustment was performed for maternal

For peer review only

age, squared maternal age, and binary variables including tobacco use, folate supplementation, past ETOP, and past abortions. Missing values for folate substitution were categorised as a separate category. Maternal ages for the missing values (n=8) were imputed using the median age of 34 years. Missing data on tobacco consumption (n=16), past ETOP (n=9), and past abortions (n=9) were imputed as 'no'.

Statistical analyses were conducted using R (version 4.3.1) and STATA version 17 (Stata Corp, College Station, Texas) [19 25].

The study protocol received approval from the ENTIS scientific committee. In most participating centres, this observational cohort study did not require ethics committee approval. However, in centres where it was necessary, appropriate ethics committee approval was obtained from the relevant authorities.

Results

In this study, we included 168 pregnant women who were exposed to a GLP1-RA during the first trimester of pregnancy. Additionally, 156 pregnant women with a diagnosis of diabetes mellitus and 163 pregnant overweight or obese were included in two distinct reference groups.

Maternal characteristics

Table 1 provides a summary of maternal characteristics, including obstetric and medical conditions. Women of the overweight/obese group were the youngest (median age 32 years) followed by those of GLP1-RA group (median age 34 years). The initial TIS contact of the diabetic group was later than the other two groups. In comparison, the GLP1-RA group had the fewest primigravid women, with the majority of women having experienced more than two previous pregnancies.

In the group exposed to GLP1-RA, a substantial proportion of patients had a BMI of 25 kg/m^2 or higher (86.6%), and 27.4% had pregestational diabetes. Of the diabetic women, 81.1% were overweight or obese. Medical conditions are summarised in Table 1. Hypertension and dyslipidemia were more

For peer review only

frequently reported in the GLP1-RA exposed and in the diabetic reference group. Psychiatric conditions were more frequently reported in the overweight/obese reference group.

Drug exposure

Liraglutide (n = 99) was the most commonly prescribed GLP1-RA followed by semaglutide (n = 51), dulaglutide (n = 11) and exenatide (n = 7). The indications for the use of GLP1-RA in the study included weight loss (n = 117, 70%), diabetes (n = 46, 28%) and other indications (n = 4, 2%) such as polycystic ovary syndrome (PCOS), dumping syndrome, or metabolic syndrome (Supplemental Figure 1). In 2022, there was a remarkable surge in the number of women receiving GLP1-RA. Therapy was initiated prior to conception in 88% of the women in the exposed group and stopped at a median gestational age of 5 weeks (IQR 4-6 weeks, minimum: 2 weeks, maximum: 40 weeks). In the GLP1-RA exposed group, concomitant or subsequent use of antidiabetic medications was reported in 44 (26.2%) cases meaning in almost all diabetic women (n=46) and of these, use of more than one (non GLP1-RA) antidiabetic medication was reported in 19 cases. Antidiabetic medications included insulin (n=21), metformin (n=19), sodium-glucose co-transporter 2 (SGLT2) inhibitors (n=5), and dipeptidyl peptidase 4 (DPP-4) inhibitors (n=1). In the diabetic reference group (n=156), all women were treated with antidiabetic medication. Use of more than one antidiabetic medication was reported in 73 (46.8%) cases. It included metformin (n=139), insulin (n=63), DPP-4 inhibitors (n=23), sulfonylureas (n=12), SGLT2 inhibitors (n=6), thiazolidinediones (n=2), and repaglinide (n=3). In the overweight/obese reference group, the use of insulin was reported for the treatment of gestational diabetes in 2 cases (1%). A higher proportion of patients in both the diabetes and the overweight/obese reference groups was exposed to multiple medications (indicating the use of any medication).

Pregnancy outcomes

Table 2 presents the proportions of offspring with major birth defects in pregnancies exposed to GLP1-RA agonists, the diabetic reference group, and the overweight/obese reference group. The rates of major birth defects, excluding genetic or chromosomal anomalies and those associated with

For peer review only

intrauterine infections, were similar in both the GLP1-RA agonist exposed group and the diabetic reference group (2.6% and 2.3%, respectively). After adjustment for maternal age, number of previous pregnancies and use of > 1 medication, the odds ratio [OR] was 0.78; 95% confidence interval [CI], 0.13–4.83. In comparison with the overweight/obese reference group, which had a rate of major birth defects of 3.9%, the adjusted OR was 0.63 (95% CI, 0.13–3.07). In the GLP1-RA agonist group, three major birth defects comprising one congenital heart defect, one congenital anomaly of the kidney, and one case of multiple anomalies were observed, suggesting that these anomalies appear to be aetiologically distinct. Supplemental Table 1 provides comprehensive information on reported anomalies, concomitant medications, and maternal conditions.

A total of 483 women were included in the cumulative incidence analysis, including 167 in the GLP1-RA agonist group, 154 in the diabetes reference group, and 162 in the overweight/obese reference group. In the GLP1-RA agonist group, cumulative incidence estimates for live births, pregnancy losses, and pregnancy terminations were 58%, 22%, and 18%, respectively (Figure 1). The corresponding estimates in the diabetes reference group were 64%, 26%, and 6%, while in the overweight/obese reference group, they were 63%, 28%, and 8%.

Details for other pregnancy and neonatal outcomes are presented in Table 3. The rate of preterm births was almost doubled in the diabetic group (15.1%) and in the overweight/obese group (14.5%) compared to the GLP1-RA group (8.0%). The rates of infants classified as LGA were higher in the GLP1-RA exposed group and in the diabetes reference group compared to the overweight/obese reference group. The Cox proportional cause-specific hazards models (Table 4) revealed no increased risk of pregnancy loss in either the comparison between the GLP1-RA agonist group and the diabetes reference group or the overweight/obese reference group. However, in the unadjusted and adjusted analysis, the GLP1-RA group showed an increased risk of termination of pregnancy (TOP) compared to the diabetes reference group (HR, 2.92; 95% CI, 1.18–7.20; p = 0.02; HRadj, 3.89; 95% CI, 1.48–10.2; p = 0.01). Nonetheless, no significant increase in the risk of TOP was observed when the GLP1-RA group

For peer review only

was compared to the overweight/obese reference group (HR, 1.79; 95% CI, 0.89–3.62; p = 0.10; HRadj, 1.39; 95% CI, 0.66–2.93; p = 0.38.

Follow-up

Information on follow-up was obtained from healthcare professionals and patients in similar proportions for both the GLP1-RA and the diabetic and overweight/obese reference groups: HCPs 10% versus 9% and 6%, patients 90% versus 91% and 94%, respectively. Infant age at follow-up was reported for 61 % in the GLP1-RA group, 73 % in the diabetes group, and 72% in the overweight/obese group. The infant age at follow-up was similar in the GLP1-RA group (neonates aged 0-28 days 8%; infants aged 28 days to 2 years 76%; children aged > 2 years 16%, and the diabetes reference group (neonates 0%; infants 63%; children 37%) and the overweight/obese reference group (neonates 4%; infants 87%; children 9%).

Discussion

This prospective multicentre observational study adds further evidence by assessing pregnancy outcomes following exposure in early pregnancy to GLP1-RA. Examining 168 pregnant women exposed to a GLP1-RA agonist during the first trimester of pregnancy, alongside two reference groups (comprising pregnant women diagnosed with diabetes mellitus and overweight/obese pregnant women), we did not identify a specific pattern of birth defects. Furthermore, our analysis revealed no association between GLP1-RA exposure and an increased risk of major birth defects. These findings align with a recently published multinational population-based cohort study. In comparison with insulin, this study also found no increased risk of major congenital malformations for infants exposed to GLP1-RA during the periconceptional period [12].

The crude rate of major birth defects in the GLP1-RA exposed women was 2.6%, aligning with the prevalence of major birth defects as reported by EUROCAT which was 2.6% including genetic anomalies for the years of 2005-2021 [27-29]. This rate is also equivalent to the rate observed in the diabetic reference group in our study (2.3%). As diabetes with poor glycaemic control is associated with an

For peer review only

increased risk of major birth defects [30], the rate of 2.3% seems low. Indeed, the rate of major birth defects often lies between 5% to 10% among women with pregestational diabetes [25 31-33] but depends on the maternal blood sugar level. There is a linear positive correlation between the risk of major congenital anomalies and pre-existing diabetes. The risk of congenital malformations in offspring of diabetic mothers generally exceeds the risk in the general population as soon as peri-conception HbA1C exceeds 6.5% [34]. Unfortunately, we lacked systematic access to glycated haemoglobin values, which could have indicated that cases in our study population were with well-managed diabetes inadvertently selected leading to a rate of birth defects similar to the general population. In addition, the low risk of TOPs in the diabetic reference group might reflect a higher proportion of planned pregnancies, benefiting from better diabetes control. We did not observe an increased risk of pregnancy losses when comparing the GLP1-RA group with the two reference groups. However, the higher incidence of elective terminations for personal reasons in the GLP1-RA group, compared to both reference groups, may be indicative of both a greater number of unplanned pregnancies and anxiety related to the unknown risks of GLP1-RA medication for the fetus.

Interestingly, 70% of the exposed women took the drug for weight reduction, although the primary drug treatment indication is diabetes type 2 as yet. As of 2018, an increasing number of women have used GLP1-RA for weight loss (see Supplemental Figure 1).

Strengths and limitations of prospective observational pregnancy cohort studies based on ENTIS data have already been described in the literature [20]. The study design with two reference groups (diabetes and overweight/obese) served to mitigate the influence of potential confounding factors. However, additional data (in particular glycated haemoglobin) would have enabled a more precise description of the severity of the disease in the patients with diabetes. Given the primary focus of our study on exposures during the first trimester, our analysis did not consider the potential risks associated with gestational diabetes. Furthermore, the treatment with GLP1-RA was discontinued during early gestation ages, in most cases. Thus, regarding cases with exposure to GLP1-RA of shorter half-lives,

For peer review only

such as liraglutide and exenatide, the exposure window did not encompass the entire first trimester of pregnancy in most cases. GLP1-RA were analysed as a homogeneous group, to explore a potential class effect. When taken individually, only a limited number of women were exposed to specific GLP1-RA such as dulaglutide and exenatide, and there were no cases of exposure to albiglutide and beinaglutide. Finally, the sample size is not large enough to draw firm and definitive conclusions.

Conclusion

This study offers further reassurance in cases of inadvertent exposure to GLP1-RA during the first trimester of pregnancy. Documentation and follow-up of these pregnancies is important to allow for further studies on a broader scale.

Author Statement

KD, SS, ODC, AP, APSW, UW contributed to study conception and design. KD, SS, CWS, MB, AH, JLR, GE, RM, ODC, UW contributed to acquisition of the data. KD, CWS, VR, LD, DH, MCA, DB, AP, ODC, FG, UW contributed to data analysis. KD, SS, CWS, MB, AH, JLR, GE, RM, VR, LD, DH, APSW, MCA, DB, AP, FL, ODC, FG, UW contributed to interpretation of data, drafting and revising the manuscript for intellectual content.

For peer review only

References

1. Lau T. Semaglutid in der Adipositastherapie: Viel Diskussionsstoff. *Dtsch Arztebl* 2023;120(33-34): A-1372 / B-1182
2. Singh GK, DiBari JN. Marked Disparities in Pre-Pregnancy Obesity and Overweight Prevalence among US Women by Race/Ethnicity, Nativity/Immigrant Status, and Sociodemographic Characteristics, 2012-2014. *J Obes* 2019;2019:2419263. doi: 10.1155/2019/2419263 [published Online First: 20190210]
3. Torkildsen SE, Svendsen H, Raisanen S, et al. Country of birth and county of residence and association with overweight and obesity-a population-based study of 219 555 pregnancies in Norway. *J Public Health (Oxf)* 2019;41(4):e290-e99. doi: 10.1093/pubmed/fdz001
4. Ryan BL, Bray Jenkyn K, Shariff SZ, et al. Beyond the grey tsunami: a cross-sectional population-based study of multimorbidity in Ontario. *Can J Public Health* 2018;109(5-6):845-54. doi: 10.17269/s41997-018-0103-0 [published Online First: 20180718]
5. Singh S, Sedgh G, Hussain R. Unintended pregnancy: worldwide levels, trends, and outcomes. *Stud Fam Plann* 2010;41(4):241-50. doi: 10.1111/j.1728-4465.2010.00250.x
6. Greco D. Normal pregnancy outcome after first-trimester exposure to liraglutide in a woman with Type 2 diabetes. *Diabet Med* 2015;32(10):e29-30. doi: 10.1111/dme.12726
7. AstraZeneca. Exenatide Pregnancy Registry. 2016 [Available from: http://clinicaltrials.gov/ct2/show/NCT00579150?cond=%22Diabetes%2C+Gestational%22&rank=22).
8. Dogan SE, Kuskonmaz SM, Koc G, et al. Case Series: Exposure to Glucagon-like Peptide-1 Receptor Agonist in the First Trimester of Pregnancy in Two Siblings. *Endocr Metab Immune Disord Drug Targets* 2023 doi: 10.2174/0118715303252109231023115112 [published Online First: 20231102]
9. Skov K, Mandic IN, Nyborg KM. Semaglutide and pregnancy. *Int J Gynaecol Obstet* 2023 doi: 10.1002/ijgo.15092 [published Online First: 20230908]
10. Alghamdi A, Alsaeddi A, Malki H, et al. A Case Report of a Pregnant Woman With Type 2 Diabetes Mellitus Using Dulaglutide During the First Trimester of Pregnancy. *Cureus* 2023;15(9):e44644. doi: 10.7759/cureus.44644 [published Online First: 20230904]
11. EMA. Ozempic: EPAR - Product information 2023 [Available from: https://www.ema.europa.eu/en/documents/product-information/ozempic-epar-product-information_en.pdf.
12. Cesta CE, Rotem R, Bateman BT, et al. Safety of GLP-1 Receptor Agonists and Other Second-Line Antidiabetics in Early Pregnancy. *JAMA Intern Med* 2023 doi: 10.1001/jamainternmed.2023.6663 [published Online First: 20231211]
13. FDA. US. Non-Clinical Review(s). Ozempic (Semaglutide). Center for Drug Evaluation and Research. 2017 [Available from: https://www.accessdata.fda.gov/drugsatfda_docs/nda/2017/209637Orig1s000Approv.pdf.
14. FDA. US. Pharmacology Review(s). Drug Approval Package, Trulicity (dulaglutide) injection. 2014 [Available from: https://www.accessdata.fda.gov/drugsatfda_docs/nda/2014/125469Orig1s000PharmR.pdf.
15. FDA. US. Victoza (Liraglutide [rDNA]) Injection. Drug Approval Package. Pharmacology Review(s). Part 1. 2008 [Available from: https://www.accessdata.fda.gov/drugsatfda_docs/nda/2010/022341s000pharmr_P1.pdf.
16. FDA. US. Byetta (Exenatide) Injection. Drug Approval Package. Pharmacology Reviews. 2004 [Available from: https://www.accessdata.fda.gov/drugsatfda_docs/nda/2009/021919s000ClinPharmR.pdf.
17. Muller DRP, Stenvers DJ, Malekzadeh A, et al. Effects of GLP-1 agonists and SGLT2 inhibitors during pregnancy and lactation on offspring outcomes: a systematic review of the evidence. *Front Endocrinol (Lausanne)* 2023;14:1215356. doi: 10.3389/fendo.2023.1215356 [published Online First: 20231010]

For peer review only

18. Tetro N, Moushaev S, Rubinchik-Stern M, et al. The Placental Barrier: the Gate and the Fate in Drug Distribution. *Pharm Res* 2018;35(4):71. doi: 10.1007/s11095-017-2286-0 [published Online First: 20180223]
19. Winterfeld U, Klinger G, Panchaud A, et al. Pregnancy outcome following maternal exposure to mirtazapine: a multicenter, prospective study. *J Clin Psychopharmacol* 2015;35(3):250-9. doi: 10.1097/JCP.0000000000000309
20. Schaefer C, Ornoy A, Clementi M, et al. Using observational cohort data for studying drug effects on pregnancy outcome--methodological considerations. *Reprod Toxicol* 2008;26(1):36-41. doi: 10.1016/j.reprotox.2008.05.064 [published Online First: 20080607]
21. Winterfeld U, Allignol A, Panchaud A, et al. Pregnancy outcome following maternal exposure to statins: a multicentre prospective study. *BJOG* 2013;120(4):463-71. doi: 10.1111/1471-0528.12066 [published Online First: 20121130]
22. Dao K, Shechtman S, Diav-Citrin O, et al. Reproductive Safety of Trazodone After Maternal Exposure in Early Pregnancy: A Comparative ENTIS Cohort Study. *J Clin Psychopharmacol* 2023;43(1):12-19. doi: 10.1097/JCP.0000000000001630
23. EUROCAT. EUROCAT Guide 1.5: Instruction for the registration of congenital anomalies. EUROCAT Central Registry, University of Ulster. [updated December 2022. Available from: https://eu-rd-platform.jrc.ec.europa.eu/eurocat_en.
24. WHO. MGRS, Multicentre Growth Reference Study, www.who.int/childgrowth/standards. 1997-2003
25. Panchaud A, Rousson V, Vial T, et al. Pregnancy outcomes in women on metformin for diabetes or other indications among those seeking teratology information services. *Br J Clin Pharmacol* 2018;84(3):568-78. doi: 10.1111/bcp.13481 [published Online First: 20180114]
26. Rousson V, Allignol A, Aurousseau A, et al. Stabilizing cumulative incidence estimation of pregnancy outcome with delayed entries. *Biom J* 2019;61(5):1290-302. doi: 10.1002/bimj.201700237 [published Online First: 20180708]
27. Sheets MTBF. Critical Periods of Development. 2021 [Available from: https://www.ncbi.nlm.nih.gov/books/NBK582659/.
28. EUROCAT. Prevalence charts and tables 2023 [Available from: https://eu-rd-platform.jrc.ec.europa.eu/eurocat/eurocat-data/prevalence_en.
29. (CDC). CfDCaP. Update on overall prevalence of major birth defects--Atlanta, Georgia, 1978-2005. *MMWR Morb Mortal Wkly Rep* 2008;57(1):1-5.
30. (NICE) NIfHaCE. Diabetes in pregnancy: management from preconception to the postnatal period 2020 [Available from: https://www.nice.org.uk/guidance/ng3/resources/diabetes-in-pregnancy-management-from-preconception-to-the-postnatal-period-pdf-51038446021.
31. Reece EA. Diabetes-induced birth defects: what do we know? What can we do? *Curr Diab Rep* 2012;12(1):24-32. doi: 10.1007/s11892-011-0251-6
32. Yang GR, Dye TD, Li D. Effects of pre-gestational diabetes mellitus and gestational diabetes mellitus on macrosomia and birth defects in Upstate New York. *Diabetes Res Clin Pract* 2019;155:107811. doi: 10.1016/j.diabres.2019.107811 [published Online First: 20190808]
33. Tinker SC, Gilboa SM, Moore CA, et al. Specific birth defects in pregnancies of women with diabetes: National Birth Defects Prevention Study, 1997-2011. *Am J Obstet Gynecol* 2020;222(2):176 e1-76 e11. doi: 10.1016/j.ajog.2019.08.028 [published Online First: 20190824]
34. Bell R, Glinianaia SV, Tennant PW, et al. Peri-conception hyperglycaemia and nephropathy are associated with risk of congenital anomaly in women with pre-existing diabetes: a population-based cohort study. *Diabetologia* 2012 doi: 10.1007/s00125-012-2455-y [published Online First: 20120208]

For peer review only

Table 1 Maternal characteristics and indication for GLP1-RA agonists use			
**Characteristics**	**GLP1 agonist group (n=168)**	**Diabetic reference group (n=156)**	**Overweight/obese reference group (n=163)**
Maternal age (years), n	166	156	158
Median (IQR)	34 (30-37)	36 (32-40)	32 (29-36)
Tobacco use, n	162	147	162
n (%)	21 (13.0)	18 (12.2)	16 (9.9)
Alcohol consumption, n	162	138	162
n (%)	3 (1.9)	2 (1.5)	8 (4.9)
GA initial contact (weeks), n	168	156	163
Median (IQR)	6 (5-9)	7 (6-12)	6 (5-9)
Previous pregnancies, n	167	152	162
0, n (%)	18 (10.8)	39 (25.7)	52 (32.1)
1, n (%)	31 (18.6)	36 (23.7)	42 (25.9)
≥2, n (%)	118 (70.7)	77 (50.7)	68 (42.0)
Previous spontaneous abortions, n	167	151	160
0, n (%)	120 (71.9)	110 (72.9)	121 (75.6)
1, n (%)	30 (18.0)	26 (17.2)	24 (15.0)
≥2, n (%)	17 (10.2)	15 (9.9)	15 (9.4)
Previous ETOP, n	167	151	160
0, n (%)	154 (92.2)	134 (88.7)	145 (90.6)
≥1, n (%)	13 (7.8)	17 (11.3)	15 (9.4)
Pregestational diabetes, n	168	156	-
n (%)	46 (27.4)	156 (100.0)[1]	-[2]
Overweight/obesity, n	164	154	161
BMI 25-30 kg/m^2, n (%)	45 (27.4)	37 (24.0)	56 (34.8)
BMI ≥ 30 kg/m^2, n (%)	97 (59.2)	88 (57.1)	103 (64.0)
Other medical conditions, n	80	147	141
Hypertension n (%)	13 (16.3)	31 (21.1)	7 (5.0)
Dyslipidemia n (%)	7 (8.8)	14 (9.5)	0 (0)
Hypothyroidism n (%)	8 (10.0)	14 (9.5)	16 (11.3)
Psychiatric condition n (%)	9 (11.3)	20 (13.6)	52 (36.9)
Epilepsy n (%)	1 (1.3)	1 (0.7)	0 (0)
Auto-immune disease n (%)	5 (6.3)	7 (4.8)	14 (9.9)
Other n (%)	24 (30.0)	36 (24.5)	54 (38.3)
Any medication in pregnancy, n	168	156	163
n (%)	102 (60.7)	139 (89.1)	121 (74.2)

n = number of patients; IQR = interquartile range; GA = gestational age; ETOP = elective termination of pregnancy

[1] inclusion criterion; [2] exclusion criterion

For peer review only

Table 2 Risk of birth defects in pregnancies exposed to GLP1-RA during the first trimester compared to the two reference groups

	GLP1-RA agonist group	Diabetic reference group	Overweight/obese reference group	GLP1-RA agonist group vs. diabetic reference group: OR (95%CI)	GLP1-RA agonist group vs. diabetic reference group: OR_{adj} (95%CI)[d]	GLP1-RA agonist group vs. overweight/obese reference group: OR (95%CI)	GLP1-RA agonist group vs. overweight/obese reference group: OR_{adj} (95%CI)[d]
Major birth defects excluding chromosomal/genetic/infection associated, n (%)	3/117 (2.6)[a]	3/128 (2.3)[b]	5/127 (3.9)[c]	1.10 (0.22-5.54)	0.78 (0.13-4.83)	0.64 (0.15-2.75)	0.63 (0.13-3.07)
Major birth defects – subgroups of anomalies							
Congenital anomalies of kidney, n	1						
Urogenital anomalies, n			1				
Congenital heart defects, n	1		2				
Nervous system anomalies, n		1	1				
Polymalformative syndrome, n	1	1					
Limb anomalies, n		1					
Gastro-intestinal anomalies, n			1				

[a] Limited to pregnancies with available data on the presence or absence of a birth defect, included 113 livebirths, 3 terminated pregnancies, and 1 stillbirth.

[b] Limited to pregnancies with available data on the presence or absence of a birth defect, included 126 livebirths, 1 terminated pregnancy, and 1 stillbirth

[c] Limited to pregnancies with available data on the presence or absence of a birth defect, included 117 livebirths, 7 terminated pregnancy, and 3 stillbirths

[d] adjusted for maternal age (continuous), number of previous pregnancies (0,1,>=2), use of > 1 medication (yes/no)

For peer review only

Table 3 Pregnancy outcomes

	GLP1-RA group (n=168)	Diabetic reference group (n=156)	Overweight/obese reference group (n=163)
Live-born infants, n	113	126	117
Elective termination of pregnancy for personal reasons, n	20	5	6
Medical termination of pregnancy, n	3[a]	1[b]	7[c,d]
Spontaneous abortion, n	31	23	29
Stillbirth, n	1	1	4
Preterm delivery, n(%) (n=113;126;127)	9 (8.0)	19 (15.1)	17 (14.5)
GA at birth (wk), median (IQR) (n=113;125;117)	39 (38-40)	38 (37-39)	39 (38-40)
Birthweight (g), median (IQR) (n=113;124;117)	3400 (3100-3660)	3370 (2890-3682)	3245 (2850-3560)
Large for gestational age, n(%) (n=113;124;117)	20 (17.7)	29 (23.4)	7 (6.0)
Small for gestational age, n(%) (n=113;124;117)	9 (8.0)	10 (8.1)	17 (14.5)
Delivery mode, n(%) (n=112;135;108)			
Spontaneous vaginal delivery	60 (53.6)	68 (50.4)	60 (55.6)
Assisted delivery and caesarean section	52 (46.4)	67 (49.6)	48 (44.4)

[a] reason for medical termination of pregnancy: 1 congenital anomaly, 1 genetic anomaly, 1 caesarean scar pregnancy. [b] reason for medical termination of pregnancy: 1 congenital anomaly. [c] reason for medical termination of pregnancy: 5 congenital anomalies (see table 2), in addition, one was due to an intrauterine CMV infection associated with anomalies, 1 genetic anomaly; [d]1 ectopic pregnancy

For peer review only

Table 4 : Pregnancy outcomes - Cox Analysis

	GLP1-RA group vs. diabetic reference group		GLP1-RA group vs. overweight/obese reference group	
	**HR (95% CI)**	**HR_{adj} (95% CI)[a]**	**HR (95% CI)**	**HR_{adj} (95% CI)[a]**
Pregnancy loss[b]	1.10 (0.65-1.89)	1.67 (0.93-3.01)	0.92 (0.56-1.49)	0.80 (0.48-1.33)
Pregnancy termination[c]	2.92 (1.18-7.20)	3.89 (1.48-10.2)	1.79 (0.89-3.62)	1.39 (0.66-2.93)

[a]adjusted for maternal age, tobacco use, folate intake, past abortion or termination history. [b] spontaneous abortions, stillbirths. [c] elective or medical termination of pregnancy

For peer review only

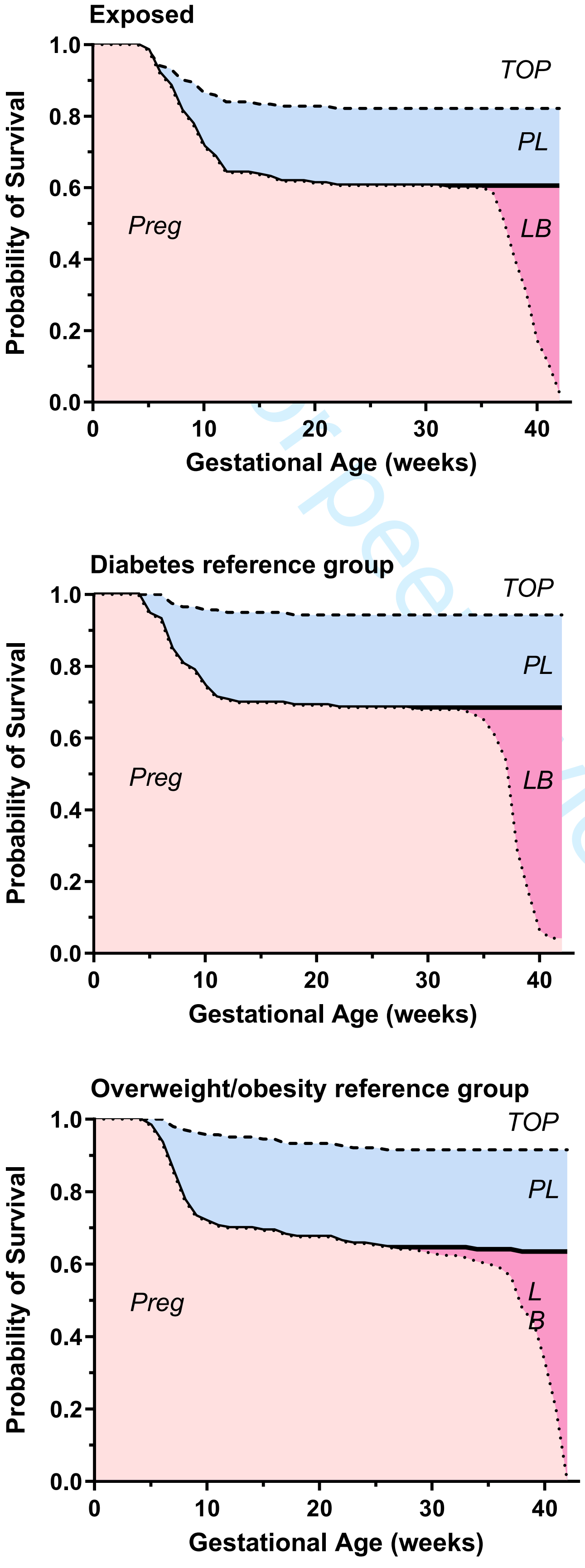
Exposed
TOP
PL
Preg
LB
Probability of Survival
1.0
0.8
0.6
0.4
0.2
0.0
0
10
20
30
40
Gestational Age (weeks)
Diabetes reference group
TOP
PL
Preg
LB
Probability of Survival
Gestational Age (weeks)
Overweight/obesity reference group
TOP
PL
Preg
L
B
Probability of Survival
Gestational Age (weeks)

Figure 1: Cumulative incidences of pregnancy outcomes with livebirth (LB), pregnancy losses (PL) and pregnancy termination (TOP) in women exposed to glucagon-like peptide 1 receptor agonists, the diabetes reference group and the overweight/obesity reference group.

For peer review only

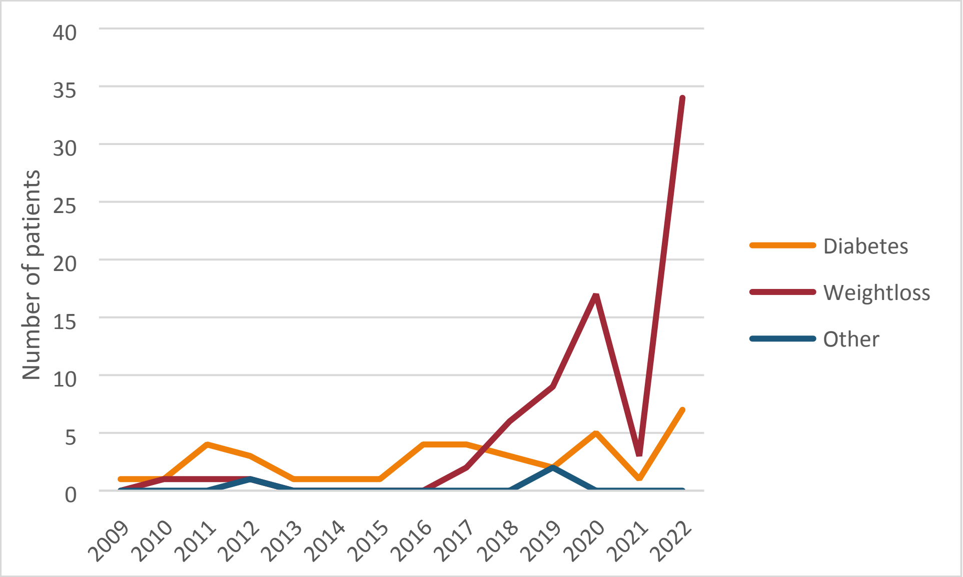

Supplemental Figure 1: Evolution of the clinical indications for glucagon-like peptide 1 receptor agonists

For peer review only

**Major birth defects in GLP1-RA exposed pregnancies**	**Outcome**	**GA at birth / pregnancy loss§**	**GLP1-RA (dose) / indication**	**Exposure§**	**Concomitant medications and pre-/pregnancy supplements (GA of exposure)**	**Maternal conditions**
Ventricular septal defect	Live birth	38	Liraglutide (unknown) / Weight loss	0-6	folate (6-12)	Obesity (BMI 34 kg/m²), gestational diabetes
Ectopic kidney	Live birth	38	Semaglutide (1 mg/wk) / Weight loss	0-4	methylphenidate (until week 6), folate (started before pregnancy)	Endometriosis, ADHD, alcohol (< 1 drink/day, timing unknown), BMI 25 kg/m²
Multiple (brain, hands and heart) anomalies (nothing was found post mortem to suggest genetic or cytogenetic finding)	TOP	17	Liraglutide (unknown) / Weight loss	0-5	folate (started before pregnancy)	Overweight (BMI 27 kg/m²)
**Major birth defects in diabetic patients pregnancies**	**Outcome**		**Antidiabetic drugs / indication**		**Concomitant medications and pre-/pregnancy supplements (GA of exposure)**	**Maternal conditions**
Club foot	Live birth	38	Metformin 1700 mg/d diabetes	0-7	folate (started before pregnancy), aluminium hydroxide*	Borderline personality, polycystic ovaries, obesity (BMI 40 kg/m²)

Supplemental Table 1 Major birth defects in GLP1-RA and the diabetic and overweight/obese reference groups, concomitant medications, and maternal conditions

For peer review only

Polymalformative syndrome (holoprosencephaly, rib deformity, corpus callosum agenesis, patent foramen ovale, hemivertebra, renal agenesis, spine malformation, ventricular septal defect, congenital disorder of glycosylation)	Live birth	40	Sitagliptin 100 mg/d, dapagliflozine, insulin diabetes	0-6<br>0-6<br>6-40	folate (started at 6 weeks), levothyroxine (0-40)	obesity (BMI 40 kg/m^2), hypothyroidism, tobacco (< 5 cig/day)
Spina bifida & other anomalies typical of diabetes	TOP	18	Metformin 2550 mg/d, glimepiride, insuline, diabetes	0-6<br>0-5<br>0-40	losartan (0-5), simvastatin (0-5)	Multiple sclerosis, arterial hypertension, obesity (BMI 34 kg/m^2)
**Major birth defects in overweight/obese patients pregnancies**	**Outcome**				**Concomitant medications**	**Maternal conditions**
Tetralogy of Fallot	Live birth	36			paracetamol (0-40), dipyrone*, passiflora valerian*, thiamine*, pyridoxine*, folate (started before pregnancy)	Overweight (BMI 28 kg/m^2)
Double outlet ventricle	TOP	17			escitalopram (0-17) acetylsalicylic acid 1000 mg*, folate (started before pregnancy), dental X-ray (6)	Obesity (BMI 30 kg/m^2), migraine
Acrania	TOP	15			sulpiride (0-15), acetazolamide oral (0-15), esomeprazole (0-15), folate (started before pregnancy)	Obesity (BMI 46 kg/m^2), schizophrenia
Biliary atresia	TOP	22			thyroxine (4-22), cefuroxime (7-8), influenza vaccination (14), folate (from beginning of pregnancy)	Obesity (BMI 36 kg/m^2), hypothyroidism
Oligohydramnios on early detailed U/S, urinary bladder distention/dilatation, compatible with posterior urethral valve	TOP	17			budesonide and formoterol (6-17), folate (started before pregnancy	Overweight (BMI 29 kg/m^2), asthma

For peer review only

* sometine in pregnancy, § weeks of gestation, GA : gestational age; TOP: termination of pregnancy						

For peer review only

Reporting checklist for cohort study.

Based on the STROBE cohort guidelines.

Instructions to authors

Complete this checklist by entering the page numbers from your manuscript where readers will find each of the items listed below.

Your article may not currently address all the items on the checklist. Please modify your text to include the missing information. If you are certain that an item does not apply, please write "n/a" and provide a short explanation.

Upload your completed checklist as an extra file when you submit to a journal.

In your methods section, say that you used the STROBE cohortreporting guidelines, and cite them as:

von Elm E, Altman DG, Egger M, Pocock SJ, Gotzsche PC, Vandenbroucke JP. The Strengthening the Reporting of Observational Studies in Epidemiology (STROBE) Statement: guidelines for reporting observational studies.

		Reporting Item	Page Number
**Title and abstract**			
Title	#1a	Indicate the study’s design with a commonly used term in the title or the abstract	1

For peer review only

Abstract	#1b	Provide in the abstract an informative and balanced summary of what was done and what was found	3
**Introduction**			
Background / rationale	#2	Explain the scientific background and rationale for the investigation being reported	4-6
Objectives	#3	State specific objectives, including any prespecified hypotheses	6
**Methods**			
Study design	#4	Present key elements of study design early in the paper	6
Setting	#5	Describe the setting, locations, and relevant dates, including periods of recruitment, exposure, follow-up, and data collection	6
Eligibility criteria	#6a	Give the eligibility criteria, and the sources and methods of selection of participants. Describe methods of follow-up.	7-8
Eligibility criteria	#6b	For matched studies, give matching criteria and number of exposed and unexposed	7-8
Variables	#7	Clearly define all outcomes, exposures, predictors, potential confounders, and effect modifiers. Give diagnostic criteria, if applicable	8-10
Data sources / measurement	#8	For each variable of interest give sources of data and details of methods of assessment (measurement). Describe comparability of assessment methods if there is more than	8-10

For peer review only

		one group. Give information separately for for exposed and unexposed groups if applicable.	
Bias	#9	Describe any efforts to address potential sources of bias	9
Study size	#10	Explain how the study size was arrived at	7
Quantitative variables	#11	Explain how quantitative variables were handled in the analyses. If applicable, describe which groupings were chosen, and why	9-10
Statistical methods	#12a	Describe all statistical methods, including those used to control for confounding: 9-10	
Statistical methods	#12b	Describe any methods used to examine subgroups and interactions	NA
Statistical methods	#12c	Explain how missing data were addressed	9-10
Statistical methods	#12d	If applicable, explain how loss to follow-up was addressed	NA
Statistical methods	#12e	Describe any sensitivity analyses: NA	

Results

Participants	#13a	Report numbers of individuals at each stage of study—eg numbers potentially eligible, examined for eligibility,	10

For peer review only

		confirmed eligible, included in the study, completing follow-up, and analysed. Give information separately for for exposed and unexposed groups if applicable.	
Participants	#13b	Give reasons for non-participation at each stage	NA
Participants	#13c	Consider use of a flow diagram	
Descriptive data	#14a	Give characteristics of study participants (eg demographic, clinical, social) and information on exposures and potential confounders. Give information separately for exposed and unexposed groups if applicable.	10-11
Descriptive data	#14b	Indicate number of participants with missing data for each variable of interest: Tables	
Descriptive data	#14c	Summarise follow-up time (eg, average and total amount): 13	
Outcome data	#15	Report numbers of outcome events or summary measures over time. Give information separately for exposed and unexposed groups if applicable.: 11-12	
Main results	#16a	Give unadjusted estimates and, if applicable, confounder-adjusted estimates and their precision (eg, 95% confidence	Tables

For peer review only

		interval). Make clear which confounders were adjusted for and why they were included	
Main results	#16b	Report category boundaries when continuous variables were categorized	Tables
Main results	#16c	If relevant, consider translating estimates of relative risk into absolute risk for a meaningful time period	
Other analyses	#17	Report other analyses done—eg analyses of subgroups and interactions, and sensitivity analyses	NA
**Discussion**			
Key results	#18	Summarise key results with reference to study objectives	12
Limitations	#19	Discuss limitations of the study, taking into account sources of potential bias or imprecision. Discuss both direction and magnitude of any potential bias.	14
Interpretation	#20	Give a cautious overall interpretation considering objectives, limitations, multiplicity of analyses, results from similar studies, and other relevant evidence.	12-15
Generalisability	#21	Discuss the generalisability (external validity) of the study results	12-17
**Other Information**			

For peer review only

Funding	#22	Give the source of funding and the role of the funders for the present study and, if applicable, for the original study on which the present article is based	1

None The STROBE checklist is distributed under the terms of the Creative Commons Attribution License CC-BY. This checklist can be completed online using https://www.goodreports.org/, a tool made by the EQUATOR Network in collaboration with Penelope.ai

For peer review only

BMJ Open

Use of GLP1 receptor agonists in early pregnancy and reproductive safety: a multicentre, observational, prospective cohort study based on the databases of six Teratology Information Services

Journal:	*BMJ Open*
Manuscript ID	bmjopen-2023-083550.R1
Article Type:	Original research
Date Submitted by the Author:	08-Mar-2024

Complete List of Authors:	Dao, Kim ; Centre Hospitalier Universitaire Vaudois (CHUV) and University of Lausanne, Swiss Teratogen Information Service and Clinical Pharmacology Service<br>Shechtman, Svetlana; Ministry of Health, The Israeli Teratology Information Service<br>Weber-Schoendorfer, Corinna; Charité Universitätsmedizin Berlin, Pharmakovigilanzzentrum Embryonaltoxikologie, Institut für Klinische Pharmakologie und Toxikologie<br>Diav-Citrin, Orna ; Ministry of Health, The Israeli Teratology Information Service; Hebrew University of Jerusalem Hadassah Medical School<br>Murad, Reem; Ministry of Health, The Israeli Teratology Information Service<br>Berlin, Maya; Shamir Medical Center Assaf Harofeh, Clinical Pharmacology and Toxicology Unit, Drug Consultation Center, Zerifin TIS, affiliated with the Faculty of Medicine, Tel Aviv University<br>Hazan, Ariela; Shamir Medical Center Assaf Harofeh, Clinical Pharmacology and Toxicology Unit, Drug Consultation Center, Zerifin TIS, affiliated with the Faculty of Medicine, Tel Aviv University<br>Richardson, Jonathan L; Newcastle Upon Tyne Hospitals NHS Foundation Trust, The UK Teratology Information Service<br>Eleftheriou, Georgios; Hospital ASST Papa Giovanni XXIII, Poison Control Center<br>Rousson, Valentin; University of Lausanne, Center for Primary Care and Public Health<br>Diezi, Leonore; Centre Hospitalier Universitaire Vaudois (CHUV) and University of Lausanne, Swiss Teratogen Information Service and Clinical Pharmacology Service<br>Haefliger, David; Centre Hospitalier Universitaire Vaudois (CHUV) and University of Lausanne, Swiss Teratogen Information Service and Clinical Pharmacology Service<br>Simões-Wüst, Ana Paula ; University Hospital Zurich, University of Zurich, Department of Obstetrics<br>Addor, Marie-Claude; Centre Hospitalier Universitaire Vaudois (CHUV), Department of Woman-Mother-Child<br>Baud, David; Centre Hospitalier Universitaire Vaudois (CHUV) and University of Lausanne, Materno-Fetal and Obstetrics Research Unit, Department Woman-Mother-Child<br>Lamine, Faiza; Centre Hospitalier Universitaire Vaudois, Endocrinology,

	Diabetes and Metabolism Service; Hôpital Riviera-Chablais, Endocrinology and Diabetes Unit. Internal Medicine Service.<br>Panchaud, Alice; University of Bern, Institute of Primary Health Care (BIHAM); Centre Hospitalier Universitaire Vaudois, Service of Pharmacy<br>Buclin, Thierry; Centre Hospitalier Universitaire Vaudois (CHUV) and University of Lausanne, Swiss Teratogen Information Service and Clinical Pharmacology Service<br>Girardin, François; Centre Hospitalier Universitaire Vaudois (CHUV) and University of Lausanne, Swiss Teratogen Information Service and Clinical Pharmacology Service<br>Winterfeld, Ursula; Centre Hospitalier Universitaire Vaudois, Swiss Teratogen Information Service and Clinical Pharmacology Service

**Primary Subject Heading**:	Pharmacology and therapeutics
Secondary Subject Heading:	Obstetrics and gynaecology
Keywords:	Pregnant Women, Safety, Diabetes in pregnancy < DIABETES & ENDOCRINOLOGY, Obesity

SCHOLARONE™
Manuscripts

I, the Submitting Author has the right to grant and does grant on behalf of all authors of the Work (as defined in the below author licence), an exclusive licence and/or a non-exclusive licence for contributions from authors who are: i) UK Crown employees; ii) where BMJ has agreed a CC-BY licence shall apply, and/or iii) in accordance with the terms applicable for US Federal Government officers or employees acting as part of their official duties; on a worldwide, perpetual, irrevocable, royalty-free basis to BMJ Publishing Group Ltd ("BMJ") its licensees and where the relevant Journal is co-owned by BMJ to the co-owners of the Journal, to publish the Work in this journal and any other BMJ products and to exploit all rights, as set out in our licence.

The Submitting Author accepts and understands that any supply made under these terms is made by BMJ to the Submitting Author unless you are acting as an employee on behalf of your employer or a postgraduate student of an affiliated institution which is paying any applicable article publishing charge ("APC") for Open Access articles. Where the Submitting Author wishes to make the Work available on an Open Access basis (and intends to pay the relevant APC), the terms of reuse of such Open Access shall be governed by a Creative Commons licence – details of these licences and which Creative Commons licence will apply to this Work are set out in our licence referred to above.

Other than as permitted in any relevant BMJ Author's Self Archiving Policies, I confirm this Work has not been accepted for publication elsewhere, is not being considered for publication elsewhere and does not duplicate material already published. I confirm all authors consent to publication of this Work and authorise the granting of this licence.

For peer review only

Use of GLP1 receptor agonists in early pregnancy and reproductive safety: a multicentre, observational, prospective cohort study based on the databases of six Teratology Information Services

Kim Dao, MD[1], Svetlana Shechtman, PhD[2], Corinna Weber-Schoendorfer, MD-PhD[3], Orna Diav-Citrin, MD[2,4], Reem Hegla Murad[2], Maya Berlin, PhD[5], Ariela Hazan[5], Jonathan Luke Richardson, PhD[6], Georgios Eleftheriou, MD-PhD[7], Valentin Rousson, PhD[8], Leonore Diezi[1], MD, David Haefliger[1], MD, Ana Paula Simoes-Wüst[9], PhD, Marie-Claude Addor, MD[10], David Baud, MD-PhD[11], Faiza Lamine[12] MD, Alice Panchaud[13,14], PhD, Thierry Buclin, MD-PhD[1], François Girardin, MD[1*], Ursula Winterfeld, PhD[1*]

[1] Swiss Teratogen Information Service and Clinical Pharmacology Service, Centre Hospitalier Universitaire Vaudois (CHUV) and University of Lausanne, Lausanne, Switzerland

[2] The Israeli Teratology Information Service, Ministry of Health, Jerusalem, Israel

[3] Charité - Universitätsmedizin Berlin, Pharmakovigilanzzentrum Embryonaltoxikologie, Institut für Klinische Pharmakologie und Toxikologie, Berlin, Germany

[4] The Hebrew University and Hadassah Medical School, Jerusalem, Israel

[5] Clinical Pharmacology and Toxicology Unit, Drug Consultation Center, Shamir Medical Center (Assaf Harofeh), Zerifin TIS, affiliated with the Faculty of Medicine, Tel Aviv University, Tel Aviv, Israel

[6] The UK Teratology Information Service, Newcastle upon Tyne Hospitals NHS Foundation Trust, Newcastle Upon Tyne, UK

[7] Poison Control Center, Hospital ASST Papa Giovanni XXIII, Bergamo, Italy

[8] Center for Primary Care and Public Health, University of Lausanne, Switzerland

[9] Department of Obstetrics, University Hospital Zurich, University of Zurich, Switzerland

[10] Department of Woman-Mother-Child, Centre Hospitalier Universitaire Vaudois (CHUV), Lausanne, Switzerland

[11] Materno-Fetal and Obstetrics Research Unit, Department Woman-Mother-Child, Centre Hospitalier Universitaire Vaudois (CHUV) and University of Lausanne, Lausanne, Switzerland

[12] Endocrinology, Diabetes and Metabolism Service. Centre Hospitalier Universitaire Vaudois (CHUV), Lausanne, Switzerland. Endocrinology and Diabetes Unit. Internal Medicine Service. Hôpital Riviera Chablais, Rennaz, Switzerland.

[13] Institute of Primary Health Care (BIHAM), University of Bern, Bern, Switzerland

[14] Service of Pharmacy, Centre Hospitalier Universitaire Vaudois (CHUV), Lausanne, Switzerland

* contributed equally

Journal: **BMJ Open** as an **Original Contribution**

Word count: 3723

For peer review only

Corresponding author
Dr. Ursula Winterfeld

Swiss Teratology Information Service (STIS) and Clinical Pharmacology Service, Lausanne University Hospital and University of Lausanne, Lausanne, Switzerland

e-mail: ursula.winterfeld@chuv.ch

Tel: +41-21-3144267

For peer review only

Abstract

Objectives: Glucagon-like peptide 1 receptor agonists (GLP1-RA) are indicated for the treatment of type 2 diabetes and more recently for weight loss. The aim of this study was to assess the risks associated with GLP1-RA exposure during early pregnancy.

Design: This multicentre, observational prospective cohort study compared pregnancy outcomes in women exposed to a GLP1-RA in early pregnancy either for diabetes or obesity treatment with those in two reference groups: (i) diabetic women exposed to at least one non-GLP1-RA antidiabetic drug during the first trimester and (ii) a reference group of overweight/obese women without diabetes, between 2009 and 2022.

Setting: Data were collected from the databases of six Teratology Information Services.

Participants: This study included 168 pregnancies of women exposed to a GLP1-RA during the first trimester, alongside a reference group of 156 pregnancies of women with diabetes and 163 pregnancies of overweight/obese women.

Results: Exposure to a GLP1-RA in the first trimester was not associated with a risk of major birth defects when compared to diabetes (2.6% versus 2.3%; adjusted odds ratio (OR) [95% confidence interval], 0.98 [0.16–5.82]) or to overweight/obese (2.6% versus 3.9%; adjusted OR 0.54 [0.11–2.75]). For the GLP1-RA group, cumulative incidence for live births, pregnancy losses, and pregnancy terminations was 59%, 23%, and 18%, respectively. In the diabetes reference group, corresponding estimates were 69%, 26%, and 6%, while in the overweight/obese reference group, they were 63%, 29%, and 8%. Cox proportional cause-specific hazards models indicated no increased risk of pregnancy losses in the GLP1-RA versus the diabetes and the overweight/obese reference groups, in both crude and adjusted analyses.

Conclusions: This study offers reassurance in cases of inadvertent exposure to GLP1-RA during the first trimester of pregnancy. Due to the limited sample size, larger studies are required to validate these findings.

For peer review only

Keywords: Glucagon-like peptide 1 receptor agonists, liraglutide, semaglutide, dulaglutide, exenatide pregnancy, reproductive safety.

Strengths and limitations of this study

- This observational prospective multicentre study aimed to assess the reproductive safety of early pregnancy exposure to GLP1-RA, providing valuable insights into a previously underexplored area.
- The study design incorporated two reference groups (diabetes and overweight/obese), to reduce the influence of potential confounding variables.
- GLP1-RA were analysed as a single homogeneous group, only allowing for the exploration of potential class effects as specific GLP1-RA had limited individual exposure.
- The availability of additional data, such as glycated haemoglobin or fasting glucose levels, varied within the study sample, which affected our ability to precisely describe disease severity among patients with diabetes.

Introduction

Glucagon-like peptide 1 receptor agonists (GLP1-RA) have been widely used as therapeutic agents for the management of type 2 diabetes. More recently, certain GLP1-RA (liraglutide, semaglutide) have also gained approval in some countries for the treatment of obesity. The demand for GLP1-RA approved for obesity is high, so that there have even been supply shortages in Europe [1]. Since the prevalence of both overweight and obesity among women of childbearing age is increasing substantially in most countries (between 30% in some European countries and nearly 50% in the USA), it is to be expected that a further growing number of women of reproductive age will be treated with a GLP1-RA [2-4]. Approximately 50% of pregnancies worldwide are unplanned [5], therefore the question of the safety of GLP1-RA is particularly relevant.

This drug class includes short acting GLP1-RA exenatide, lixisenatide and beinaglutide, as well as long-acting GLP1-RA dulaglutide, semaglutide, liraglutide and albiglutide (discontinued in 2017 at the request of the marketing-authorisation holder due to steady decline in sales). By mimicking the actions

For peer review only

of the hormone glucagon-like peptide 1, these agents improve blood glycaemic control by increasing glucose-dependent insulin secretion, decreasing inappropriate glucagon secretion, and regulate appetite (by increasing satiety and delaying gastric emptying) to promote weight loss. The safety profile of GLP1-RA use during the first trimester of pregnancy has been investigated in previous studies, which did not reveal an elevated risk of major birth defects. One single case of exposure to liraglutide in the first trimester has been reported, describing a favourable outcome for the newborn [6]. A registry for exenatide documented seven cases of exposure during pregnancy, but follow-up information is lacking [7]. A case report describes the outcomes of two separate pregnancies in a 40-year-old woman with diabetes and obesity undergoing exenatide treatment. The report details the birth of one healthy child and another pregnancy resulting in a child with an atrial septal defect, which spontaneously resolved by the age of three [8]. One single case of exposure to semaglutide in early pregnancy due to off-label treatment for polycystic ovary syndrome was reported with no birth defect [9]. Another case of exposure to dulaglutide during first trimester of pregnancy for the treatment of type 2 diabetes did not result in any reported birth defects [10]. Due to paucity of data concerning semaglutide and human pregnancy outcomes, the manufacturer advises stopping this treatment two months before conception [11]. In a multinational population-based cohort study involving 51’826 pregnant women diagnosed with type 2 diabetes and their infants, the standardized prevalence of major congenital malformations was 8.3% among infants with periconceptional exposure to GLP1-RA (n = 938) [12]. Compared with insulin, there was no increased risk of major congenital malformations (adjusted RR 0.95 95% CI, 0.72-1.26) for infants exposed to GLP1-RA.

According to product labelling, animal studies have indicated a potential for reproductive toxicity at maternally toxic doses for semaglutide, dulaglutide, exenatide and liraglutide. An increased risk of malformations was observed for liraglutide and semaglutide at doses comparable to those administered in humans [13-17]. GLP1-RA are characterised by their high molar mass, ranging from 3700 g/mol (liraglutide) to 4100 g/mol (semaglutide and exenatide) and even up to 63,000 g/mol

For peer review only

(dulaglutide). Even though additional physico-chemical properties play important roles, placental transfer of drugs with such high molecular size is generally not anticipated in the first trimester of pregnancy, unless a specific mechanism for transfer exists [18].

While previous studies did not detect an increased risk of major birth defects following the use of GLP1-RA during the first trimester of pregnancy, further confirmation from additional studies is warranted. The primary objective of this study was to prospectively evaluate the risk of major birth defects, spontaneous pregnancy losses (including abortions and stillbirths) and pregnancy terminations following first-trimester exposure to a GLP1-RA. Secondary objectives included describing additional pregnancy outcomes, such as gestational weeks at birth, birth weight, and categorical neonatal outcomes like preterm birth and small or large for gestational age.

Material & Methods

Study design and dataset description

This multicentre, prospective, observational cohort study was conducted involving six participating centres that are members of the European Network of Teratology Information Services (ENTIS) in five countries: Germany, Israel, Italy, Switzerland, and the United Kingdom. ENTIS is an organisation of specialised services providing expertise on potential risks associated with medication exposure during pregnancy and breastfeeding at an individual level [19]. Standardised protocols are followed for data collection at each participating centre [20]. Methodological aspects pertaining to collaborative studies of a similar nature have been extensively documented in the existing literature, thus serving as a reference for performing the present study [21].

Health professionals and pregnant women spontaneously contact a Teratology Information Service (TIS) for a risk assessment during pregnancy. Data collection is performed at this first contact with the TIS and following the anticipated date of delivery. Standardised questionnaires are used, administered to the patient and/or their health-care provider. Maternal characteristics, such as age, tobacco use

For peer review only

and alcohol consumption, as well as medical and obstetric history, are recorded. Detailed information regarding drug exposure and drug treatment indication, including dose, timing of therapy initiation, duration and concurrent medications, is also documented during the initial contact with the TIS. After the expected date of delivery, follow-up is conducted through structured mailed questionnaires and/or a structured telephone interview. The follow-up process seeks information on various aspects, including further medication use, pregnancy outcomes, gestational age at delivery, birth weight, presence of birth defects, and neonatal complications. For this study, additional data pertaining to diabetes severity and obesity were also collected, where available. Socioeconomic and educational data were mostly not recorded [22].

Exposed and reference groups

The study sample was comprised of women exposed to any GLP1-RA, and two reference groups: one of diabetic women, the other of overweight/obese women without diabetes. The patients, or their healthcare providers, contacted one of the six participating TIS between 2009 and 2022. Only patients with prospectively ascertained pregnancy outcomes, meaning those with unknown pregnancy outcomes or prenatal pathologies at the time of study enrolment, were included. The GLP1-RA exposed group consisted of pregnant women who used at least one GLP1-RA (identified by ATC codes A10BJ, A10AE54 or A10AE56) either as monotherapy or in combination with other medications during the first trimester of pregnancy.

The first reference group included pregnancies of patients diagnosed with pre-existing diabetes mellitus (ICD-10 codes E10-E13), who were exposed to non-GLP1-RA antidiabetic drugs during the first trimester of pregnancy (in most cases metformin). Patient selection for this reference group was done through a randomised process, choosing from the same prospective cohort within the corresponding TIS and within the same time frame of contact (±3 year). The second reference group primarily included pregnancies of patients classified as overweight (BMI ≥ 25 kg/m^2) or obese (BMI ≥ 30 kg/m^2) (ICD-10 code E66). They were aimed to be matched for BMI with the exposed group for a BMI of ± 2 kg/m^2.

For peer review only

Similarly, patient selection within this reference group was randomised from the same prospective cohort within the corresponding TIS and within the same time frame of contact (±3 year).

Exclusion criteria for all three groups included exposure to any teratogenic drugs such as systemic retinoids (including acitretin, alitretinoin, bexarotene, isotretinoin, tretinoin), cytotoxic agents, selected antiepileptic drugs (including valproate, carbamazepine, phenytoin, fosphenytoin, primidone, topiramate, phenobarbital), thalidomide, leflunomide, lenalidomide, coumarin derivatives (including dicoumarol, phenindione, warfarin, phenprocoumon, acenocoumarol, fluindione), lithium, misoprostol, carbimazole, and methimazole/thiamazole at any time during pregnancy. Additionally, exclusion criteria encompassed exposure to angiotensin-converting enzyme inhibitors, angiotensin II receptor blockers, or tetracyclines during the second and third trimesters, presence of malignancies or malignancy-related conditions, multiple pregnancies, and duplicate cases. Data sent from different TIS were analysed anonymously [22].

Outcomes

Birth defects were classified by two independent co-authors (MCA, DB) who were blinded to exposure data. The classification was performed using the European Network of Population-based Registries for the Epidemiological Surveillance of Congenital Anomalies (EUROCAT) ICD10-BPA system [23]. The assessment of major birth defects was restricted to live births and pregnancy losses with confirmed outcomes after appropriate medical examination, ensuring reliable knowledge on birth defect status. Birth defects with genetic or chromosomal anomalies and those occurring due to intrauterine infections were excluded. Due to significant likelihood of underreporting, minor birth defects were not included in the evaluation. Primary outcomes also included the risks of spontaneous pregnancy losses, which comprised both abortions and stillbirths as a combined outcome, categorised based on gestational age as either $\leq$22 weeks or >22 weeks, respectively. Additionally, the risks of pregnancy terminations were analysed. Secondary outcomes were preterm birth (<37 weeks of gestation after LMP), gestational age at birth and birth weight. Large (LGA) and small (SGA) for gestational age were

For peer review only

also compared between cohorts. LGA was defined as a birthweight exceeding the 90th percentile, and SGA as birthweight less than the 10th percentile based on WHO infant sex-specific growth charts [24].

Statistical Analysis

Crude risks of major birth defects were determined by dividing the total number of infants or fetuses with birth defects by the sum of all live-born infants, plus the number of cases with known birth defects in stillbirths and terminated pregnancies [19]. To assess the association between GLP1-RA exposure and the risk of major birth defects, multivariate logistic regression analysis was conducted, generating an odds ratio (OR) with a corresponding 95% confidence interval (CI) and adjusted for confounding factors, including maternal age (≤ 35, > 35 years), number of previous pregnancies and polymedication [19]. The number of previous pregnancies was categorised (0, 1, ≥2), and polymedication was defined as the use of more than one drug, including any other medication. Adjustment for potential confounding factors was done for covariates that were imbalanced between groups. An additional category for missing values was included to account for instances where data on any of the confounding factors, such as maternal age, history of previous pregnancies and polymedication, were not available [7]. In addition to the primary analysis, we conducted a sensitivity analysis to include all reported birth defects, irrespective of aetiology including those of genetic or chromosomal origin and those occurring due to intrauterine infections.

Elective pregnancy terminations for personal reasons (ETOP) and elective terminations for medical reasons (MTOP), as well as spontaneous pregnancy losses (miscarriages and stillbirths), were treated as competing events in this study. The frequency of these outcomes was represented using cumulative incidence functions [25], while delayed entries were treated as described by Rousson et al. [26]. Women were thus considered to be at risk of experiencing one of these outcomes solely from their gestational age at the time of contact with the TIS. This allowed us to take into account the gestational age at the time of TIS contact, acknowledging that it was not independent of the outcome, given that certain pregnancy terminations may not be feasible at an advanced gestational age. Cases with missing

For peer review only

information on gestational age at call or pregnancy outcome were excluded from the cumulative incidence analysis. To assess the association between exposure and the overall risk of pregnancy loss, cause-specific Cox proportional hazards models were used, considering any variation in gestational age at enrolment across the three groups. In the hazard models, adjustment was performed for maternal age, squared maternal age, and binary variables including tobacco use, folate supplementation, past ETOP, and past abortions. Missing values for folate substitution were categorised as a separate category. Maternal ages for the missing values (n=8) were imputed using the median age of 34 years. Missing data on tobacco consumption (n=16), past ETOP (n=9), and past abortions (n=9) were imputed as 'no'.

Statistical analyses were conducted using R (version 4.3.1) and STATA version 17 (Stata Corp, College Station, Texas) [19, 25].

The study protocol received approval from the ENTIS scientific committee. In most participating centres, this observational cohort study did not require ethics committee approval. However, in centres where it was necessary, appropriate ethics committee approval was obtained from the relevant authorities.

Patient and public involvement

Patients and/or the public were not involved in the design, or conduct, or reporting, or dissemination plans of this research.

Results

In this study, we included 168 pregnant women who were exposed to a GLP1-RA during the first trimester of pregnancy. Additionally, 156 pregnant women with a diagnosis of diabetes mellitus and 163 pregnant overweight or obese were included in two distinct reference groups.

Maternal characteristics

For peer review only

Table 1 provides a summary of maternal characteristics, including obstetric and medical conditions. Women of the overweight/obese group were the youngest (median age 32 years) followed by those of GLP1-RA group (median age 34 years). The initial TIS contact of the diabetic group was later than the other two groups. In comparison, the GLP1-RA group had the fewest primigravid women, with the majority of women having experienced more than two previous pregnancies.

In the group exposed to GLP1-RA, a substantial proportion of patients had a BMI of 25 kg/m^2 or higher (86.6%), and 27.4% had pregestational diabetes. Of the diabetic women, 81.1% were overweight or obese. Medical conditions are summarised in Table 1. Hypertension and dyslipidemia were more frequently reported in the GLP1-RA exposed and in the diabetic reference group. Psychiatric conditions were more frequently reported in the overweight/obese reference group.

Drug exposure

Liraglutide (n = 99) was the most commonly prescribed GLP1-RA followed by semaglutide (n = 51), dulaglutide (n = 11) and exenatide (n = 7). The indications for the use of GLP1-RA in the study included weight loss (n = 117, 70%), diabetes (n = 46, 28%) and other indications (n = 4, 2%) such as polycystic ovary syndrome (PCOS), dumping syndrome, or metabolic syndrome (Supplemental Figure 1). In 2022, there was a remarkable surge in the number of women receiving GLP1-RA. Therapy was initiated prior to conception in 88% of the women in the exposed group and stopped at a median gestational age of 5 weeks (IQR 4-6 weeks, minimum: 2 weeks, maximum: 40 weeks). In the GLP1-RA exposed group, concomitant or subsequent use of antidiabetic medications was reported in 44 (26.2%) cases meaning in almost all diabetic women (n=46) and of these, use of more than one (non GLP1-RA) antidiabetic medication was reported in 19 cases. Antidiabetic medications included insulin (n=21), metformin (n=19), sodium-glucose co-transporter 2 (SGLT2) inhibitors (n=5), and dipeptidyl peptidase 4 (DPP-4) inhibitors (n=1). In the diabetic reference group (n=156), all women were treated with antidiabetic medication. Use of more than one antidiabetic medication was reported in 73 (46.8%) cases. It included metformin (n=139), insulin (n=63), DPP-4 inhibitors (n=23), sulfonylureas (n=12), SGLT2

For peer review only

inhibitors (n=6), thiazolidinediones (n=2), and repaglinide (n=3). In the overweight/obese reference group, the use of insulin was reported for the treatment of gestational diabetes in 2 cases (1%). A higher proportion of patients in both the diabetes and the overweight/obese reference groups was exposed to multiple medications (indicating the use of any medication).

Pregnancy outcomes

Table 2 presents the proportions of offspring with major birth defects in pregnancies exposed to GLP1-RA agonists, the diabetic reference group, and the overweight/obese reference group. The rates of major birth defects, excluding genetic or chromosomal anomalies and those associated with intrauterine infections, were similar in both the GLP1-RA exposed group and the diabetic reference group (2.6% and 2.3%, respectively). After adjustment for maternal age, number of previous pregnancies and use of > 1 medication, the odds ratio [OR] was 0.98; 95% confidence interval [CI], 0.16–5.82. In comparison with the overweight/obese reference group, which had a rate of major birth defects of 3.9%, the adjusted OR was 0.54 (95% CI, 0.11–2.75). In the GLP1-RA group, three major birth defects comprising one congenital heart defect, one congenital anomaly of the kidney, and one case of multiple anomalies were observed, suggesting that these anomalies appear to be aetiologically distinct. Supplemental Table 1 provides comprehensive information on reported anomalies, concomitant medications, and maternal conditions. In the sensitivity analysis, the rates of major birth defects, when all major anomalies irrespective of aetiology were included, were 3.1% in the GLP1-RA exposed group and 4.2% in the diabetic reference group. The crude OR between the GLP1-RA exposed group and the diabetic reference group was 1.37 (95% CI, 0.36-5.23), and the adjusted OR was 0.99 (95% CI, 0.22-4.42). Compared to the overweight/obese reference group, which had a rate of major birth defects irrespective of aetiology of 6.3%, the crude OR was 0.66 (95% CI, 0.21-2.07) and the adjusted OR was 0.60 (95% CI, 0.16-2.21).

A total of 483 women were included in the cumulative incidence analysis, including 167 in the GLP1-RA group, 154 in the diabetes reference group, and 162 in the overweight/obese reference group. In

For peer review only

the GLP1-RA group, cumulative incidence estimates for live births, pregnancy losses, and pregnancy terminations were 59%, 23%, and 18%, respectively (Figure 1). The corresponding estimates in the diabetes reference group were 69%, 26%, and 6%, while in the overweight/obese reference group, they were 63%, 29%, and 8%.

Details for other pregnancy and neonatal outcomes are presented in Table 3. The rate of preterm births was almost doubled in the diabetic group (15.1%) and in the overweight/obese group (14.5%) compared to the GLP1-RA group (8.0%). The rates of infants classified as LGA were higher in the GLP1-RA exposed group and in the diabetes reference group compared to the overweight/obese reference group. The Cox proportional cause-specific hazards models (Table 4) revealed no increased risk of pregnancy loss in either the comparison between the GLP1-RA group and the diabetes reference group or the overweight/obese reference group. However, in the unadjusted and adjusted analysis, the GLP1-RA group showed an increased risk of termination of pregnancy (TOP) compared to the diabetes reference group (HR, 2.92; 95% CI, 1.18–7.20; p = 0.02; HRadj, 3.89; 95% CI, 1.48–10.2; p = 0.01). Nonetheless, no significant increase in the risk of TOP was observed when the GLP1-RA group was compared to the overweight/obese reference group (HR, 1.79; 95% CI, 0.89–3.62; p = 0.10; HRadj, 1.39; 95% CI, 0.66–2.93; p = 0.38.

Follow-up

Information on follow-up was obtained from healthcare professionals and patients in similar proportions for both the GLP1-RA and the diabetic and overweight/obese reference groups: healthcare professionalss 10% versus 9% and 6%, patients 90% versus 91% and 94%, respectively. Infant age at follow-up was reported for 61 % in the GLP1-RA group, 73 % in the diabetes group, and 72% in the overweight/obese group. The infant age at follow-up was similar in the GLP1-RA group (neonates aged 0-28 days 8%; infants aged 28 days to 2 years 76%; children aged > 2 years 16%, and the diabetes reference group (neonates 0%; infants 63%; children 37%) and the overweight/obese reference group (neonates 4%; infants 87%; children 9%).

Discussion

This prospective multicentre observational study adds further evidence by assessing pregnancy outcomes following exposure in early pregnancy to GLP1-RA. Examining 168 pregnant women exposed to a GLP1-RA during the first trimester of pregnancy, alongside two reference groups (comprising pregnant women diagnosed with diabetes mellitus and overweight/obese pregnant women), we did not identify a specific pattern of birth defects. Furthermore, our analysis revealed no association between GLP1-RA exposure and an increased risk of major birth defects. These findings align with a recently published multinational population-based cohort study. In comparison with insulin, this study also found no increased risk of major congenital malformations for infants exposed to GLP1-RA during the periconceptional period [12].

The crude rate of major birth defects in the GLP1-RA exposed women was 2.6%, aligning with the prevalence of major birth defects as reported by EUROCAT which was 2.6% including genetic anomalies for the years of 2005-2021 [27-29]. This rate is also equivalent to that observed in the diabetic reference group in our study (2.3%). As diabetes with poor glycaemic control is associated with an increased risk of major birth defects [30], the rate of 2.3% seems low. Indeed, the rate of major birth defects often lies between 5% to 10% among women with pregestational diabetes [25, 31-33] but depends on the maternal blood sugar level. There is a linear positive correlation between the risk of major congenital anomalies and pre-existing diabetes. The risk of congenital malformations in offspring of diabetic mothers generally exceeds the risk in the general population as soon as peri-conception HbA1C exceeds 6.5% [34]. Unfortunately, we lacked systematic access to glycated haemoglobin values, which could have indicated that cases in our study population were with well-managed diabetes inadvertently selected leading to a rate of birth defects similar to the general population. In addition, the low risk of TOPs in the diabetic reference group might reflect a higher proportion of planned pregnancies, benefiting from better diabetes control. We did not observe an increased risk of pregnancy losses when comparing the GLP1-RA group with the two reference groups.

For peer review only

However, the higher incidence of elective terminations for personal reasons in the GLP1-RA group, compared to both reference groups, may be indicative of both a greater number of unplanned pregnancies and anxiety related to the unknown risks of GLP1-RA medication for the fetus.

Interestingly, 70% of the exposed women took the drug for weight reduction, although the primary drug treatment indication remains diabetes type 2. As of 2018, an increasing number of women have used GLP1-RA for weight loss (see Supplemental Figure 1).

Strengths and limitations of prospective observational pregnancy cohort studies based on ENTIS data have already been described in the literature [20]. The study design with two reference groups (diabetes and overweight/obese) served to mitigate the influence of potential confounding factors. However, additional data (in particular glycated haemoglobin) would have enabled a more precise description of the severity of the disease in the patients with diabetes. Given the primary focus of our study on exposures during the first trimester, our analysis did not consider the potential risks associated with gestational diabetes. Furthermore, the treatment with GLP1-RA was discontinued during early gestation ages, in most cases. Thus, regarding cases with exposure to GLP1-RA of shorter half-lives, such as liraglutide and exenatide, the exposure window did not encompass the entire first trimester of pregnancy in most cases. GLP1-RA were analysed as a homogeneous group, to explore a potential class effect. When taken individually, only a limited number of women were exposed to specific GLP1-RA such as dulaglutide and exenatide, and there were no cases of exposure to albiglutide and beinaglutide. Finally, the sample size is not large enough to draw firm and definitive conclusions.

Conclusion

This study offers further reassurance in cases of inadvertent exposure to GLP1-RA during the first trimester of pregnancy. Documentation and follow-up of these pregnancies is important to allow for further studies on a broader scale.

For peer review only

Acknowledgement: The authors would like to thank pharmacists Sarit Carmi and Shira Shalit, from the Division of Clinical Pharmacy, Institute for Drug Research, School of Pharmacy, Hebrew University and Hadassah Medical school, Jerusalem, Israel, for their valuable contribution to the data collection, done while acquiring their PharmD degree. We thank the whole team at STIS for their support, in particular the computer scientist Mr. F. Veuve.

Author contributions

KD, SS, ODC, AP, APSW, UW contributed to study conception and design. KD, SS, CWS, MB, AH, JLR, GE, RM, ODC, UW contributed to acquisition of the data. KD, CWS, VR, LD, DH, MCA, DB, AP, ODC, TB, FG, UW contributed to data analysis. KD, SS, CWS, MB, AH, JLR, GE, RM, VR, LD, DH, APSW, MCA, DB, AP, FL, ODC, TB, FG, UW contributed to interpretation of data, drafting and revising the manuscript for intellectual content.

Competing interests

None declared

Funding

This work was supported by Swiss National Science Foundation grant number SNSF 320030_214844

Data sharing statement

Source data related to the cohort study are available on https://zenodo.org

Ethics approval

This study involves human participants and was approved by the Institutional review board of Charité - Universitätsmedizin Berlin (Ref: EA2/041/23), Ministry of Health Jerusalem (Ref: MOH-072-2023) and (Ref: ASF-0036-23) Shamir Medical Center. In the other centers (STIS Lausanne, UK Teratology Information Service, Newcastle upon Tyne and Poison Control Center Bergamo) no ethics approval was needed.

References

1. Lau, T., *Semaglutid in der Adipositastherapie: Viel Diskussionsstoff.* Dtsch Arztebl, 2023. **120(33-34): A-1372 / B-1182**.
2. Singh, G.K. and J.N. DiBari, *Marked Disparities in Pre-Pregnancy Obesity and Overweight Prevalence among US Women by Race/Ethnicity, Nativity/Immigrant Status, and Sociodemographic Characteristics, 2012-2014.* J Obes, 2019. **2019**: p. 2419263.
3. Torkildsen, S.E., et al., *Country of birth and county of residence and association with overweight and obesity-a population-based study of 219 555 pregnancies in Norway.* J Public Health (Oxf), 2019. **41**(4): p. e290-e299.
4. Ryan, B.L., et al., *Beyond the grey tsunami: a cross-sectional population-based study of multimorbidity in Ontario.* Can J Public Health, 2018. **109**(5-6): p. 845-854.

5. Singh, S., G. Sedgh, and R. Hussain, *Unintended pregnancy: worldwide levels, trends, and outcomes.* Stud Fam Plann, 2010. **41**(4): p. 241-50.
6. Greco, D., *Normal pregnancy outcome after first-trimester exposure to liraglutide in a woman with Type 2 diabetes.* Diabet Med, 2015. **32**(10): p. e29-30.
[dataset] 7. AstraZeneca. *Exenatide Pregnancy Registry.* 2016; Available from: http://clinicaltrials.gov/ct2/show/NCT00579150?cond=%22Diabetes%2C+Gestational%22&rank=22).
8. Dogan, S.E., et al., *Case Series: Exposure to Glucagon-like Peptide-1 Receptor Agonist in the First Trimester of Pregnancy in Two Siblings.* Endocr Metab Immune Disord Drug Targets, 2023.
9. Skov, K., I.N. Mandic, and K.M. Nyborg, *Semaglutide and pregnancy.* Int J Gynaecol Obstet, 2023.
10. Alghamdi, A., et al., *A Case Report of a Pregnant Woman With Type 2 Diabetes Mellitus Using Dulaglutide During the First Trimester of Pregnancy.* Cureus, 2023. **15**(9): p. e44644.
11. EMA. *Ozempic: EPAR - Product information*. 2023; Available from: https://www.ema.europa.eu/en/documents/product-information/ozempic-epar-product-information_en.pdf.
12. Cesta, C.E., et al., *Safety of GLP-1 Receptor Agonists and Other Second-Line Antidiabetics in Early Pregnancy.* JAMA Intern Med, 2023.
13. FDA., U.S. *Non-Clinical Review(s). Ozempic (Semaglutide). Center for Drug Evaluation and Research.* 2017; Available from: https://www.accessdata.fda.gov/drugsatfda_docs/nda/2017/209637Orig1s000Approv.pdf.
14. FDA., U.S. *Pharmacology Review(s). Drug Approval Package, Trulicity (dulaglutide) injection.* 2014; Available from: https://www.accessdata.fda.gov/drugsatfda_docs/nda/2014/125469Orig1s000PharmR.pdf.
15. FDA., U.S. *Victoza (Liraglutide [rDNA]) Injection. Drug Approval Package. Pharmacology Review(s). Part 1.* 2008; Available from: https://www.accessdata.fda.gov/drugsatfda_docs/nda/2010/022341s000pharmr_P1.pdf.
16. FDA., U.S. *Byetta (Exenatide) Injection. Drug Approval Package. Pharmacology Reviews.* 2004; Available from: https://www.accessdata.fda.gov/drugsatfda_docs/nda/2009/021919s000ClinPharmR.pdf.
17. Muller, D.R.P., et al., *Effects of GLP-1 agonists and SGLT2 inhibitors during pregnancy and lactation on offspring outcomes: a systematic review of the evidence.* Front Endocrinol (Lausanne), 2023. **14**: p. 1215356.
18. Tetro, N., et al., *The Placental Barrier: the Gate and the Fate in Drug Distribution.* Pharm Res, 2018. **35**(4): p. 71.
19. Winterfeld, U., et al., *Pregnancy outcome following maternal exposure to mirtazapine: a multicenter, prospective study.* J Clin Psychopharmacol, 2015. **35**(3): p. 250-9.
20. Schaefer, C., et al., *Using observational cohort data for studying drug effects on pregnancy outcome--methodological considerations.* Reprod Toxicol, 2008. **26**(1): p. 36-41.
21. Winterfeld, U., et al., *Pregnancy outcome following maternal exposure to statins: a multicentre prospective study.* BJOG, 2013. **120**(4): p. 463-71.
22. Dao, K., et al., *Reproductive Safety of Trazodone After Maternal Exposure in Early Pregnancy: A Comparative ENTIS Cohort Study.* J Clin Psychopharmacol, 2023. **43**(1): p. 12-19.
23. EUROCAT. *EUROCAT Guide 1.5: Instruction for the registration of congenital anomalies. EUROCAT Central Registry, University of Ulster.* December 2022; Available from: https://eu-rd-platform.jrc.ec.europa.eu/eurocat_en.
24. WHO, *MGRS, Multicentre Growth Reference Study, www.who.int/childgrowth/standards.* 1997-2003.
25. Panchaud, A., et al., *Pregnancy outcomes in women on metformin for diabetes or other indications among those seeking teratology information services.* Br J Clin Pharmacol, 2018. **84**(3): p. 568-578.

For peer review only

26. Rousson, V., et al., *Stabilizing cumulative incidence estimation of pregnancy outcome with delayed entries.* Biom J, 2019. **61**(5): p. 1290-1302.
27. Sheets, M.T.B.F. *Critical Periods of Development.* . 2021; Available from: https://www.ncbi.nlm.nih.gov/books/NBK582659/.
28. EUROCAT. *Prevalence charts and tables*. 2023; Available from: https://eu-rd-platform.jrc.ec.europa.eu/eurocat/eurocat-data/prevalence_en.
29. (CDC)., C.f.D.C.a.P., *Update on overall prevalence of major birth defects--Atlanta, Georgia, 1978-2005.* MMWR Morb Mortal Wkly Rep. , 2008. **57(1)**: p. 1-5.
30. (NICE), N.I.f.H.a.C.E. *Diabetes in pregnancy: management from preconception to the postnatal period*. 2020; Available from: https://www.nice.org.uk/guidance/ng3/resources/diabetes-in-pregnancy-management-from-preconception-to-the-postnatal-period-pdf-51038446021.
31. Reece, E.A., *Diabetes-induced birth defects: what do we know? What can we do?* Curr Diab Rep, 2012. **12**(1): p. 24-32.
32. Yang, G.R., T.D. Dye, and D. Li, *Effects of pre-gestational diabetes mellitus and gestational diabetes mellitus on macrosomia and birth defects in Upstate New York.* Diabetes Res Clin Pract, 2019. **155**: p. 107811.
33. Tinker, S.C., et al., *Specific birth defects in pregnancies of women with diabetes: National Birth Defects Prevention Study, 1997-2011.* Am J Obstet Gynecol, 2020. **222**(2): p. 176 e1-176 e11.
34. Bell, R., et al., *Peri-conception hyperglycaemia and nephropathy are associated with risk of congenital anomaly in women with pre-existing diabetes: a population-based cohort study.* Diabetologia, 2012.

For peer review only

Figure Legend:

Figure 1 - Cumulative incidences of pregnancy outcomes with livebirth (LB), pregnancy losses (PL) and pregnancy termination (TOP) in women exposed to glucagon-like peptide 1 receptor agonists, the diabetes reference group and the overweight/obesity reference group.

For peer review only

Table 1 Maternal/pregnancy characteristics in study groups			
**Characteristics**	**GLP1 agonist group (n=168)**	**Diabetic reference group (n=156)**	**Overweight/obese reference group (n=163)**
Maternal age (years), n	166	156	158
≤ 35, n (%)	102 (61.5)	72 (46.2)	107 (67.7)
> 35, n(%)	64 (38.6)	84 (53.9)	51 (32.3)
Tobacco use, n	162	147	162

Yes, n (%)	21 (13.0)	18 (12.2)	16 (9.9)
Alcohol consumption, n	162	138	162
Yes, n (%)	3 (1.9)	2 (1.5)	8 (4.9)
GA initial contact (weeks), n	168	156	163
Median (IQR)	6 (5-9)	7 (6-12)	6 (5-9)
Previous pregnancies, n	167	152	162
0, n (%)	18 (10.8)	39 (25.7)	52 (32.1)
1, n (%)	31 (18.6)	36 (23.7)	42 (25.9)
≥2, n (%)	118 (70.7)	77 (50.7)	68 (42.0)
Previous spontaneous abortions, n	167	151	160
0, n (%)	120 (71.9)	110 (72.9)	121 (75.6)
1, n (%)	30 (18.0)	26 (17.2)	24 (15.0)
≥2, n (%)	17 (10.2)	15 (9.9)	15 (9.4)
Previous ETOP, n	167	151	160
0, n (%)	154 (92.2)	134 (88.7)	145 (90.6)
≥1, n (%)	13 (7.8)	17 (11.3)	15 (9.4)
Pregestational diabetes, n	168	156	-
n (%)	46 (27.4)	156 (100.0)[1]	-[2]
Overweight/obesity, n	164	154	161
BMI 25-30 kg/m^2, n (%)	45 (27.4)	37 (24.0)	56 (34.8)
BMI ≥ 30 kg/m^2, n (%)	97 (59.2)	88 (57.1)	103 (64.0)
Other medical conditions, n	80	147	141
Hypertension n (%)	13 (16.3)	31 (21.1)	7 (5.0)
Dyslipidemia n (%)	7 (8.8)	14 (9.5)	0 (0)
Hypothyroidism n (%)	8 (10.0)	14 (9.5)	16 (11.3)
Psychiatric condition n (%)	9 (11.3)	20 (13.6)	52 (36.9)
Epilepsy n (%)	1 (1.3)	1 (0.7)	0 (0)
Auto-immune disease n (%)	5 (6.3)	7 (4.8)	14 (9.9)
Other n (%)	24 (30.0)	36 (24.5)	54 (38.3)
Any medication in pregnancy, n	168	156	163
n (%)	102 (60.7)	139 (89.1)	121 (74.2)

n = number of patients; IQR = interquartile range; GA = gestational age; ETOP = elective termination of pregnancy
[1] inclusion criterion; [2] exclusion criterion

For peer review only

Table 2 Risk of birth defects in pregnancies exposed to GLP1-RA during the first trimester compared to the two reference groups

	GLP1-RA agonist group	Diabetic reference group	Overweight/obese reference group	OR (95%CI)	OR_{adj} (95%CI)[d]	OR (95%CI)	OR_{adj} (95%CI)[d]
				GLP1-RA agonist group vs. diabetic reference group		GLP1-RA agonist group vs. overweight/obese reference group	
Major birth defects excluding chromosomal/genetic/infection associated, n (%)	3/117 (2.6)[a]	3/128 (2.3)[b]	5/127 (3.9)[c]	1.10 (0.22-5.54)	0.98 (0.16-5.82)	0.64 (0.15-2.75)	0.54 (0.11-2.75)
Major birth defects – subgroups of anomalies							
Congenital anomalies of kidney, n	1						
Urogenital anomalies, n			1				
Congenital heart defects, n	1		2				
Nervous system anomalies, n		1	1				
Polymalformative syndrome, n	1	1					
Limb anomalies, n		1					
Gastro-intestinal anomalies, n			1				

[a] Limited to pregnancies with available data on the presence or absence of a birth defect, included 113 livebirths, 3 terminated pregnancies, and 1 stillbirth.

[b] Limited to pregnancies with available data on the presence or absence of a birth defect, included 126 livebirths, 1 terminated pregnancy, and 1 stillbirth

[c] Limited to pregnancies with available data on the presence or absence of a birth defect, included 117 livebirths, 7 terminated pregnancy, and 3 stillbirths

[d] adjusted for maternal age (≤ 35, > 35 years), number of previous pregnancies (0,1,≥2), use of > 1 medication (yes/no)

For peer review only

Table 3 Pregnancy outcomes

	GLP1-RA group (n=168)	Diabetic reference group (n=156)	Overweight/obese reference group (n=163)
Live-born infants, n	113	126	117
Elective termination of pregnancy for personal reasons, n	20	5	6
Medical termination of pregnancy, n	3[a]	1[b]	7[c,d]
Spontaneous abortion, n	31	23	29
Stillbirth, n	1	1	4
Preterm delivery, n(%) (n=113;126;127)	9 (8.0)	19 (15.1)	17 (14.5)
GA at birth (wk), median (IQR) (n=113;125;117)	39 (38-40)	38 (37-39)	39 (38-40)
Birthweight (g), median (IQR) (n=113;124;117)	3400 (3100-3660)	3370 (2890-3682)	3245 (2850-3560)
Large for gestational age, n(%) (n=113;124;117)	20 (17.7)	29 (23.4)	7 (6.0)
Small for gestational age, n(%) (n=113;124;117)	9 (8.0)	10 (8.1)	17 (14.5)
Delivery mode, n(%) (n=112;135;108)			
Spontaneous vaginal delivery	60 (53.6)	68 (50.4)	60 (55.6)
Assisted delivery and caesarean section	52 (46.4)	67 (49.6)	48 (44.4)

[a] reason for medical termination of pregnancy: 1 congenital anomaly, 1 genetic anomaly, 1 caesarean scar pregnancy. [b] reason for medical termination of pregnancy: 1 congenital anomaly. [c] reason for medical termination of pregnancy: 5 congenital anomalies (see table 2), in addition, one was due to an intrauterine CMV infection associated with anomalies, 1 genetic anomaly; [d]1 ectopic pregnancy

For peer review only

Table 4 : Pregnancy outcomes - Cox proportional hazards models

	GLP1-RA group vs. diabetic reference group		GLP1-RA group vs. overweight/obese reference group	
	HR (95% CI)	HR_{adj} (95% CI)[a]	HR (95% CI)	HR_{adj} (95% CI)[a]
Pregnancy loss[b]	1.10 (0.65-1.89)	1.67 (0.93-3.01)	0.92 (0.56-1.49)	0.80 (0.48-1.33)
Pregnancy termination[c]	2.92 (1.18-7.20)	3.89 (1.48-10.2)	1.79 (0.89-3.62)	1.39 (0.66-2.93)

[a]adjusted for maternal age, tobacco use, folate intake, past abortion or termination history. [b] spontaneous abortions, stillbirths. [c] elective or medical termination of pregnancy

For peer review only

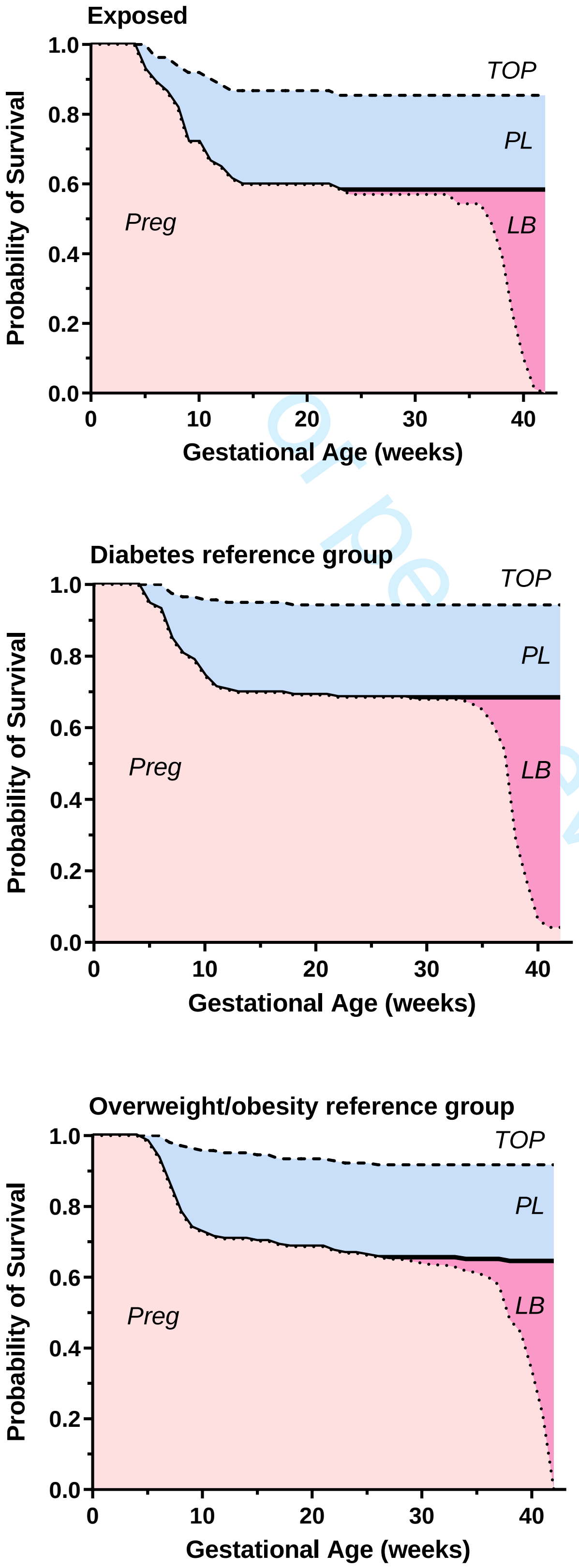

For peer review only

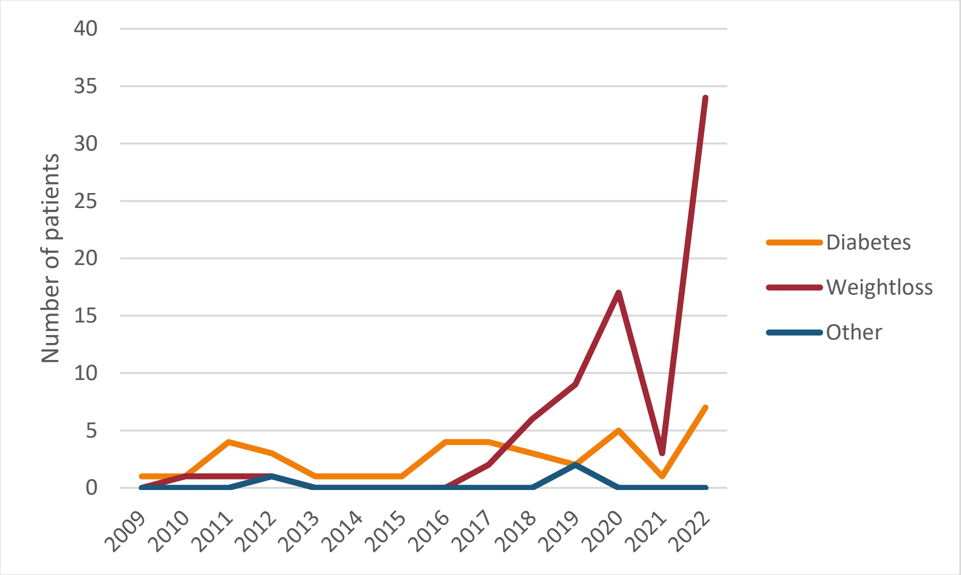

Supplemental Figure 1: Evolution of the clinical indications for glucagon-like peptide 1 receptor agonists

For peer review only

Supplemental Table 1 Major birth defects in GLP1-RA and the diabetic and overweight/obese reference groups, concomitant medications, and maternal conditions

For peer review only

Major birth defects in GLP1-RA exposed pregnancies	Outcome	GA at birth / pregnancy loss§	GLP1-RA (dose) / indication	Exposure§	Concomitant medications and pre-/pregnancy supplements (GA of exposure)	Maternal conditions
Ventricular septal defect	Live birth	38	Liraglutide (unknown) / Weight loss	0-6	folate (6-12)	Obesity (BMI 34 kg/m^2), gestational diabetes
Ectopic kidney	Live birth	38	Semaglutide (1 mg/wk) / Weight loss	0-4	methylphenidate (until week 6), folate (started before pregnancy)	Endometriosis, ADHD, alcohol (< 1 drink/day, timing unknown), BMI 25 kg/m^2
Multiple (brain, hands and heart) anomalies (nothing was found post mortem to suggest genetic or cytogenetic finding)	TOP	17	Liraglutide (unknown) / Weight loss	0-5	folate (started before pregnancy)	Overweight (BMI 27 kg/m^2)
**Major birth defects in diabetic patients pregnancies**	**Outcome**		**Antidiabetic drugs / indication**		**Concomitant medications and pre-/pregnancy supplements (GA of exposure)**	**Maternal conditions**
Club foot	Live birth	38	Metformin 1700 mg/d diabetes	0-7	folate (started before pregnancy), aluminium hydroxide*	Borderline personality, polycystic ovaries, obesity (BMI 40 kg/m^2)
Polymalformative syndrome (holoprosencephaly, rib deformity, corpus callosum agenesis, patent foramen ovale, hemivertebra, renal agenesis, spine malformation, ventricular septal defect, congenital disorder of glycosylation)	Live birth	40	Sitagliptin 100 mg/d, dapagliflozine, insulin diabetes	0-6<br>0-6<br>6-40	folate (started at 6 weeks), levothyroxine (0-40)	obesity (BMI 40 kg/m^2), hypothyroidism, tobacco (< 5 cig/day)
Spina bifida & other anomalies typical of diabetes	TOP	18	Metformin 2550 mg/d, glimepiride, insuline, diabetes	0-6<br>0-5<br>0-40	losartan (0-5), simvastatin (0-5)	Multiple sclerosis, arterial hypertensio

For peer review only

						n, obesity (BMI 34 kg/m^2)
**Major birth defects in overweight/obese patients pregnancies**	**Outc ome**				**Concomitant medications**	**Maternal conditions**
Tetralogy of Fallot	Live birth	36			paracetamol (0-40), dipyrone*, passiflora valerian*, thiamine*, pyridoxine*, folate (started before pregnancy)	Overweight (BMI 28 kg/m^2)
Double outlet ventricle	TOP	17			escitalopram (0-17) acetylsalicylic acid 1000 mg*, folate (started before pregnancy), dental X-ray (6)	Obesity (BMI 30 kg/m^2), migraine
Acrania	TOP	15			sulpiride (0-15), acetazolamide oral (0-15), esomeprazole (0-15), folate (started before pregnancy)	Obesity (BMI 46 kg/m^2), schizophreni a
Biliary atresia	TOP	22			thyroxine (4-22), cefuroxime (7-8), influenza vaccination (14), folate (from beginning of pregnancy)	Obesity (BMI 36 kg/m^2), hypothyroid ism
Oligohydramnios on early detailed U/S, urinary bladder distention/dilatation, compatible with posterior urethral valve	TOP	17			budesonide and formoterol (6-17), folate (started before pregnancy	Overweight (BMI 29 kg/m^2), asthma
* sometime in pregnancy, § weeks of gestation, GA : gestational age; TOP: termination of pregnancy						

For peer review only

Reporting checklist for cohort study.

Based on the STROBE cohort guidelines.

Instructions to authors

Complete this checklist by entering the page numbers from your manuscript where readers will find each of the items listed below.

Your article may not currently address all the items on the checklist. Please modify your text to include the missing information. If you are certain that an item does not apply, please write "n/a" and provide a short explanation.

Upload your completed checklist as an extra file when you submit to a journal.

In your methods section, say that you used the STROBE cohortreporting guidelines, and cite them as:

von Elm E, Altman DG, Egger M, Pocock SJ, Gotzsche PC, Vandenbroucke JP. The Strengthening the Reporting of Observational Studies in Epidemiology (STROBE) Statement: guidelines for reporting observational studies.

For peer review only

		Reporting Item	Page Number
**Title and abstract**			
Title	#1a	Indicate the study's design with a commonly used term in the title or the abstract	1

Abstract	#1b	Provide in the abstract an informative and balanced summary of what was done and what was found	3
**Introduction**			
Background / rationale	#2	Explain the scientific background and rationale for the investigation being reported	4-6
Objectives	#3	State specific objectives, including any prespecified hypotheses	6
**Methods**			
Study design	#4	Present key elements of study design early in the paper	6
Setting	#5	Describe the setting, locations, and relevant dates, including periods of recruitment, exposure, follow-up, and data collection	6
Eligibility criteria	#6a	Give the eligibility criteria, and the sources and methods of selection of participants. Describe methods of follow-up.	7-8
Eligibility criteria	#6b	For matched studies, give matching criteria and number of exposed and unexposed	7-8
Variables	#7	Clearly define all outcomes, exposures, predictors, potential confounders, and effect modifiers. Give diagnostic criteria, if applicable	8-10
Data sources / measurement	#8	For each variable of interest give sources of data and details of methods of assessment (measurement). Describe comparability of assessment methods if there is more than	8-10

For peer review only

		one group. Give information separately for for exposed and unexposed groups if applicable.	
Bias	#9	Describe any efforts to address potential sources of bias	9
Study size	#10	Explain how the study size was arrived at	7
Quantitative variables	#11	Explain how quantitative variables were handled in the analyses. If applicable, describe which groupings were chosen, and why	9-10
Statistical methods	#12a	Describe all statistical methods, including those used to control for confounding: 9-10	
Statistical methods	#12b	Describe any methods used to examine subgroups and interactions	NA
Statistical methods	#12c	Explain how missing data were addressed	9-10
Statistical methods	#12d	If applicable, explain how loss to follow-up was addressed	NA
Statistical methods	#12e	Describe any sensitivity analyses: NA	

Results

Participants	#13a	Report numbers of individuals at each stage of study—eg numbers potentially eligible, examined for eligibility,	10

For peer review only

		confirmed eligible, included in the study, completing follow-up, and analysed. Give information separately for for exposed and unexposed groups if applicable.	
Participants	#13b	Give reasons for non-participation at each stage	NA
Participants	#13c	Consider use of a flow diagram	
Descriptive data	#14a	Give characteristics of study participants (eg demographic, clinical, social) and information on exposures and potential confounders. Give information separately for exposed and unexposed groups if applicable.	10-11
Descriptive data	#14b	Indicate number of participants with missing data for each variable of interest: Tables	
Descriptive data	#14c	Summarise follow-up time (eg, average and total amount): 13	
Outcome data	#15	Report numbers of outcome events or summary measures over time. Give information separately for exposed and unexposed groups if applicable.: 11-12	
Main results	#16a	Give unadjusted estimates and, if applicable, confounder-adjusted estimates and their precision (eg, 95% confidence	Tables

For peer review only

		interval). Make clear which confounders were adjusted for and why they were included	
Main results	#16b	Report category boundaries when continuous variables were categorized	Tables
Main results	#16c	If relevant, consider translating estimates of relative risk into absolute risk for a meaningful time period	
Other analyses	#17	Report other analyses done—eg analyses of subgroups and interactions, and sensitivity analyses	NA
**Discussion**			
Key results	#18	Summarise key results with reference to study objectives	12
Limitations	#19	Discuss limitations of the study, taking into account sources of potential bias or imprecision. Discuss both direction and magnitude of any potential bias.	14
Interpretation	#20	Give a cautious overall interpretation considering objectives, limitations, multiplicity of analyses, results from similar studies, and other relevant evidence.	12-15
Generalisability	#21	Discuss the generalisability (external validity) of the study results	12-17
**Other Information**			

For peer review only

Funding	#22	Give the source of funding and the role of the funders for the present study and, if applicable, for the original study on which the present article is based	1

None The STROBE checklist is distributed under the terms of the Creative Commons Attribution License CC-BY. This checklist can be completed online using https://www.goodreports.org/, a tool made by the EQUATOR Network in collaboration with Penelope.ai

For peer review only
